# Supplementary material for: RoCK and ROI: single-cell transcriptomics with multiplexed enrichment of selected transcripts and region-specific sequencing
Source: Nat Commun. 2025 Dec 10;16:10991. doi: 10.1038/s41467-025-66248-z (PMC12695968; doi:10.1038/s41467-025-66248-z)
Supplement: Supplementary file 1 — Supplementary Information [file 41467_2025_66248_MOESM1_ESM.pdf]

Supplementary Information

RoCK and ROI: Single-cell transcriptomics with multiplexed enrichment of selected transcripts and region-specific sequencing

Giulia Moro[1,*], Izaskun Mallona[1,2,*,✉], Malwine J. Barz[3,4], Joël Maillard[1], Michael David Brügger[1], Hassan Fazilaty[1], Quentin Szabo[1], Tomas Valenta[1,5], Kristina Handler[6], Fiona Kerlin[7,8], Lorenz Bastian[3,4], Claudia D. Baldus[3,4], Andreas E. Moor[9], Robert Zinzen[10], Mark D. Robinson[1,2], Erich Brunner[1,✉] & Konrad Basler[1]

1. Department of Molecular Life Sciences, University of Zurich, Zurich, Switzerland
2. SIB Swiss Institute of Bioinformatics, University of Zurich, Zurich, Switzerland
3. Medical Department II, Hematology and Oncology, University Hospital Schleswig-Holstein, Kiel, Germany
4. Clinical Research Unit "CATCH ALL" (KFO 5010/1), Kiel, Germany
5. Laboratory of Cell and Developmental Biology, Institute of Molecular Genetics of the Czech Academy of Sciences, Prague, Czech Republic
6. Institute of Experimental Immunology, University of Zurich, Zurich, Switzerland
7. Berlin Institute for Medical Systems Biology (BIMSB), Max Delbrück Center for Molecular Medicine (MDC) in the Helmholtz Association, Berlin, Germany
8. Institute of Biology, Department of Biology, Chemistry, Pharmacy, Free University Berlin, Berlin, Germany
9. Department of Biosystems Science and Engineering, ETH Zürich, Basel, Switzerland
10. Systems Biology Imaging Technology Platform, Berlin Institute for Medical Systems Biology (BIMSB), Max Delbrück Center for Molecular Medicine (MDC) in the Helmholtz Association, Berlin, Germany

* Equal contribution, ✉Correspondence (erich.brunner@mls.uzh.ch, izaskun.mallona@gmail.com)

This file contains the following information:

Supplementary Note 1: Additional text on scRNA-seq experiment performed on HeLa cells (exploring detection limits)

Supplementary Figures 1-21

Supplementary Tables 1-11

Supplementary Note 1: Experiment performed on HeLa cells

To assess our method's lower detection limit, we targeted a set of transcripts expressed at less than one mRNA per cell on average, and only in a subset of cells. To determine which transcripts to target, we used a previously published bDNA sm-FISH dataset[1] which quantified transcript levels per cell on two replicates of HeLa cells, targeting 928 genes involved in various cellular functions, with probes against absent bacterial transcripts serving as negative controls.

To choose a set of lowly expressed genes for RoCK and ROI targeting, we first performed an unmodified BD Rhapsody experiment (Supp Figure 9a, Supp Figure 10a-c). The scRNA-seq run was performed on the same HeLa cells as Battich et al., 2013[1], which we used after six passages. We sequenced our library with a higher read depth than the previous experiments (over a billion reads). Since Battich et al., 2013 provide a detailed comparison of bDNA sm-FISH and bulk RNA-seq, we focused on comparing our results to theirs. We analyzed 857 genes consistently detected above bacterial background in both replicates. As expected, the BD Rhapsody results showed lower sensitivity than bDNA sm-FISH even at a high read depth (Supp Figure 9b-d). Nonetheless, using unmodified beads, the standard workflow detected extremely lowly expressed genes (e.g., *HOXA1* at 0.0428 transcripts per cell in <3% of cells; Supp Figure 11a-b,), indicating detectability is not solely determined by expression level.

By comparing the Battich et al., 2013 results and our unmodified BD Rhapsody run, we selected seven genes to target with RoCK and ROI: *RAB4B, FOXP3, KRT23, SCN9A, ADRA1D, EHD3,* and *SOX9*. Their mRNA levels were reported to range from 0.43 to 3.17 spots (transcripts) per cell (bDNA sm-FISH) on average (Supp Figure 9e-g). *RAB4B* and *FOXP3* were detected in our unmodified (untargeted) scRNA-seq and in the bulk RNA-seq performed by Battich and colleagues, while *SCN9A* and *ADRA1D* were found in the bulk RNA-seq only. To carry out a RoCK and ROI scRNA-seq experiment, we combined a 1:1 WTA:TSO library ratio using modified beads with a single capture sequence and a single ROIseq primer for each target and sequencing the library at a depth of over a billion reads (Supp Figure 10d-f, Supp Figure 12a-b). During analysis and to make library sizes comparable across the RoCK and ROI and BD Rhapsody experiments, we downsampled the latter at the level of the FASTQ file (see Methods, Supp Figure 10g-i). *RAB4B* and *FOXP3* were enriched for in the RoCK and ROI scRNA-seq experiment (Supp Figure 12c-f). A single alignment was detected for *KRT23, SCN9A* and *ADRA1D* (Supp Figure 12c). *EHD3* and *SOX9* remained undetected in the RoCK and ROI experiment (Supp 12c).

This experiment allowed us to probe the limits of RoCK and ROI detection, showing that this method can slightly improve the detection of very lowly expressed genes (e.g., *FOXP3* at 0.22 copies per cell, Supp Figure 12), while others remained poorly detectable or undetected. To improve target detection, multiple RoCK and ROI primer pairs should be tested to optimize capture and priming sites. Notably, bDNA sm-FISH signals for the targeted transcripts were minimally above the detection threshold, suggesting extremely low expression in the cells used for scRNA-seq. In addition, and consistent to our results using BD Rhapsody, only four out of the seven targets were detected even by bulk RNA-seq, underscoring the challenge of reliably detecting single-copy transcripts with current RNA-seq methods.

Supplementary Figures

Supplementary Figure 1: Sequence information on BD Rhapsody barcoded beads and size of RoCKseq modified beads

Supplementary Figure 2: Design of splints for RoCKseq bead modification

Supplementary Figure 3: Validation of RoCKseq bead modification

Supplementary Figure 4: Comparison of RoCK and ROI and standard BD Rhapsody library generation

Supplementary Figure 5: Structure of transgenic construct in cell lines, quality control metrics for scRNA-seq experiment to test addition of T primer and position of RoCK and ROI primers in constructs

Supplementary Figure 6: Quality control metrics on WTA data for scRNA-seq experiment using mix of cell lines to test RoCK and ROI performance

Supplementary Figure 7: Comparison of transcriptome for scRNA-seq experiment using mix of cell lines to test RoCK and ROI performance

Supplementary Figure 8: Analysis of target data from scRNA-seq experiment using mix of cell lines to test RoCK and ROI performance and effect of cell area on quantification of *eGFP* mRNAs

Supplementary Figure 9: Analysis of unmodified condition performed on HeLa cells

Supplementary Figure 10: Quality control on unmodified and modified conditions performed on HeLa cells

Supplementary Figure 11: Heatmaps on transcript detection in scRNA-seq compared to bDNA sm-FISH

Supplementary Figure 12: Analysis of modified experiment performed on HeLa cells

Supplementary Figure 13: Characterization of TSO data from RoCK and ROI experiments

Supplementary Figure 14: Flow of reads during RoCK and ROI scRNA-seq human and mouse mixing experiments

Supplementary Figure 15: *Pdgfrα* locus, capture, regions of interest and bead modification

Supplementary Figure 16: Cell gating for murine colonic cells

Supplementary Figure 17: Quality control of *Pdgfrα* scRNA-seq experiment, descriptive analysis and splicing quantification

Supplementary Figure 18: Detection of *Pdgfrα* alternative splicing

Supplementary Figure 19: Detection of the *BCR::ABL1* fusion transcript in leukemia cells

Supplementary Figure 20: Analysis of artificially primed products (APPs)

Supplementary Figure 21: Comparison of *BCR::ABL1* positive and negative monocytes

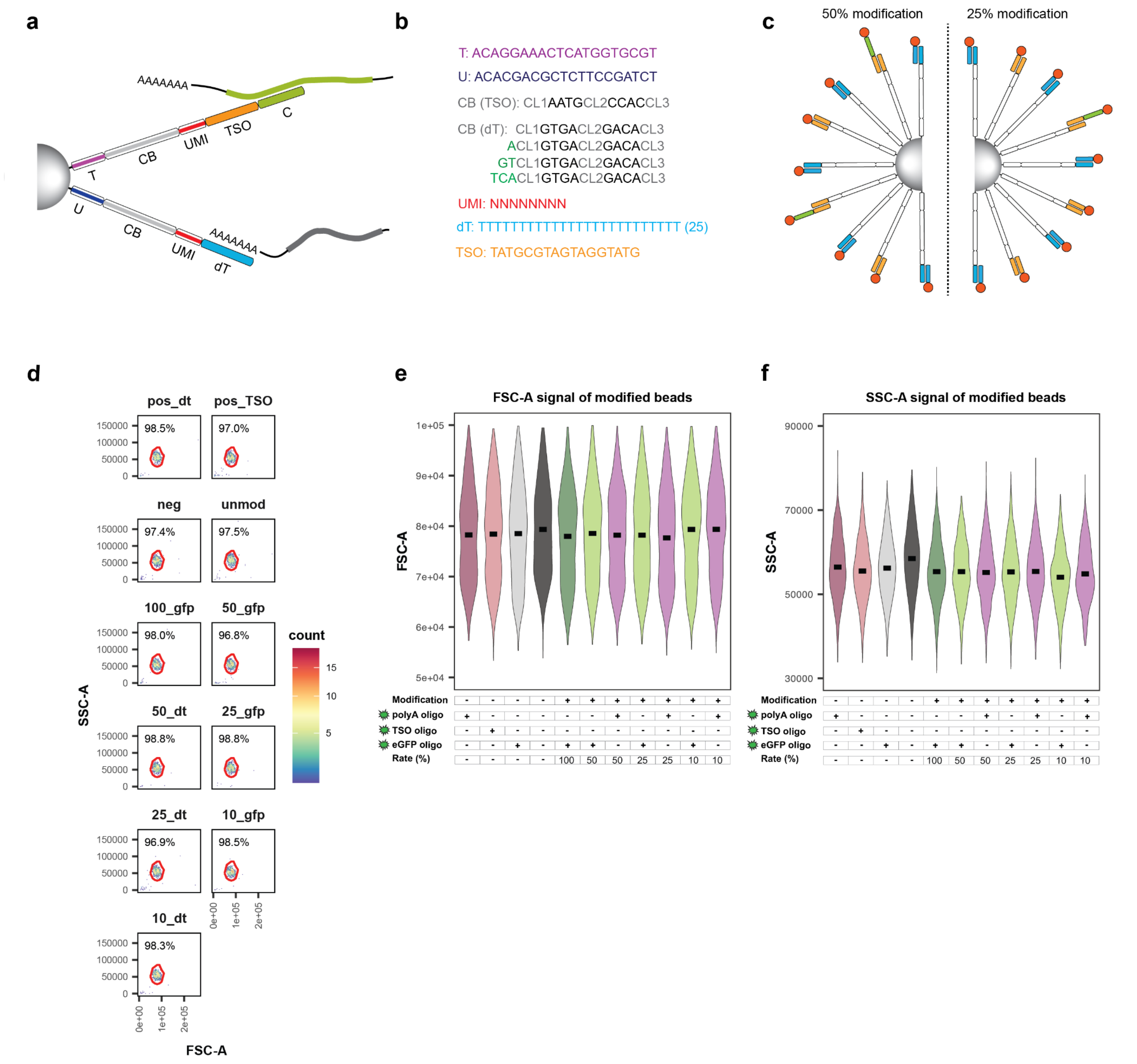

Supp Figure 1: Sequence information on BD Rhapsody barcoded beads and size of RoCKseq modified beads

a, RoCKseq BD Rhapsody beads. T: T primer, U: universal primer, CB: cell barcode, UMI: unique molecular identifier, TSO: template switching oligo, C: capture sequence added to the beads. **b,** Bead elements' sequence**:** T and Universal primers (U primers); CB (TSO): cell barcodes on TSO oligos; CB (dT): cell barcodes on WTA oligos; UMI; dT (WTA oligos); TSO (TSO oligos). **c,** Titration of RoCKseq modification; left 50% modification, right: 25% modification. The TSO titration oligo is also 5' phosphorylated (red circle) as it requires removal by the lambda exonuclease. **d-f,** Size of barcoded beads after titration of RoCKseq modification**. For panels (e-f)**: The Y-axis has a biexponential transformation.

a

cDNA sequence *eGFP*

GAAGCTTGGGCTGCAGGTCGACTCTAGAGGATCCCCGGGTACCGGTCGCCACCATGGTGAGCAAGGGCGAGGAGCTGTTCACCGGGGTGGTGCCCATCCTGGTCGAGCTGGACGG
CGACGTAAACGGCCACAAGTTCAGCGTGTCCGGCGAGGGCGAGGGCGATGCCACCTACGGCAAGCTGACCCTGAAGTTCATCTGCACCACCGGCAAGCTGCCCGTGCCCTGGCCC
ACCCTCGTGACCACCCTGACCTACGGCGTGCAGTGCTTCAGCCGCTACCCCGACCACATGAAGCAGCACGACTTCTTCAAGTCCGCCATGCCCGAAGGCTACGTCCAGGAGCGCAC
CATCTTCTTCAAGGACGACGGCAACTACAAGACCCGCGCCGAGGTGAAGTTCGAGGGCGACACCCTGGTGAACCGCATCGAGCTGAAGGGCATCGACTTCAAGGAGGACGGCAACA
TCCTGGGGCACAAGCTGGAGTACAACTACAACAGCCACAACGTCTATATCATGGCCGACAAGCAGAAGAACGGCATCAAGGTGAACTTCAAGATCCGCCACAACATCGAGGACGGCA
GCGTGCAGCTCGCCGACCACTACCAGCAGAACACCCCCATCGGCGACGGCCCCGTGCTGCTGCCCGACAACCACTACCTGAGCACCCAGTCCGCCCTGAGCAAAGACCCCAACGA
GAAGCGCGATCACATGGTCCTGCTGGAGTTCGTGACCGCCGCCGGGATCACTCTCGGCATGGACGAGCTGTACAAGTAAGCGGCCGCGACTCTAGAATTCGATATCAAGCTTATCGAT
AATCAACCTCTGGATTACAAAATTTGTGAAAGATTGACTGGTATTCTTAACTATGTTGCTCCTTTTACGCTATGTGGATACGCTGCTTTAATGCCTTTGTATCATGCTATTGCTTCCCGTATGG
CTTTCATTTTCTCCTCCTTGTATAAATCCTGGTTGCTGTCTCTTTATGAGGAGTTGTGGCCCGTTGTCAGGCAACGTGGCGTGGTGTGCACTGTGTTTGCTGACGCAACCCCCACTGGTT
GGGGCATTGCCACCACCTGTCAGCTCCTTTCCGGGACTTTCGCTTTCCCCCTCCCTATTGCCACGGCGGAACTCATCGCCGCCTGCCTTGCCCGCTGCTGGACAGGGGCTCGGCTGT
TGGGCACTGACAATTCCGTGGTGTTGTCGGGGAAATCATCGTCCTTTCCTTGGCTGCTCGCCTGTGTTGCCACCTGGATTCTGCGCGGGACGTCCTTCTGCTACGTCCCTTCGGCCCT
CAATCCAGCGGACCTTCCTTCCCGCGGCCTGCTGCCGGCTCTGCGGCCTCTTCCGCGTCTTCGCCTTCGCCCTCAGACGAGTCGGATCTCCCTTTGGGCCGCCTCCCCGCATCGATA
CCGTCGACCTCGAGACCTAGAAAAACATGGAGCAATCACAAGTAGCAATACAGCAGCTACCAATGCTGATTGTGCCTGGCTAGAAGCACAAGAGGAGGAGGAGGTGGGTTTTCCAGTC
ACACCTCAGGTACCTTTAAGACCAATGACTTACAAGGCAGCTGTAGATCTTAGCCACTTTTTAAAAGAAAAGGGGGGACTGGAAGGGCTAATTCACTCCCAACGAAGACAAGATATCCTT
GATCTGTGGATCTACCACACACAAGGCTACTTCCCTGATTGGCAGAACTACACACCAGGGCCAGGGATCAGATATCCACTGACCTTTGGATGGTGCTACAAGCTAGTACCAGTTGAGCA
AGAGAAGGTAGAAGAAGCCAATGAAGGAGAGAACACCCGCTTGTTACACCCTGTGAGCCTGCATGGGATGGATGACCCGGAGAGAGAAGTATTAGAGTGGAGGTTTGACAGCCGCCT
AGCATTTCATCACATGGCCCGAGAGCTGCATCCGGACTGTACT

b

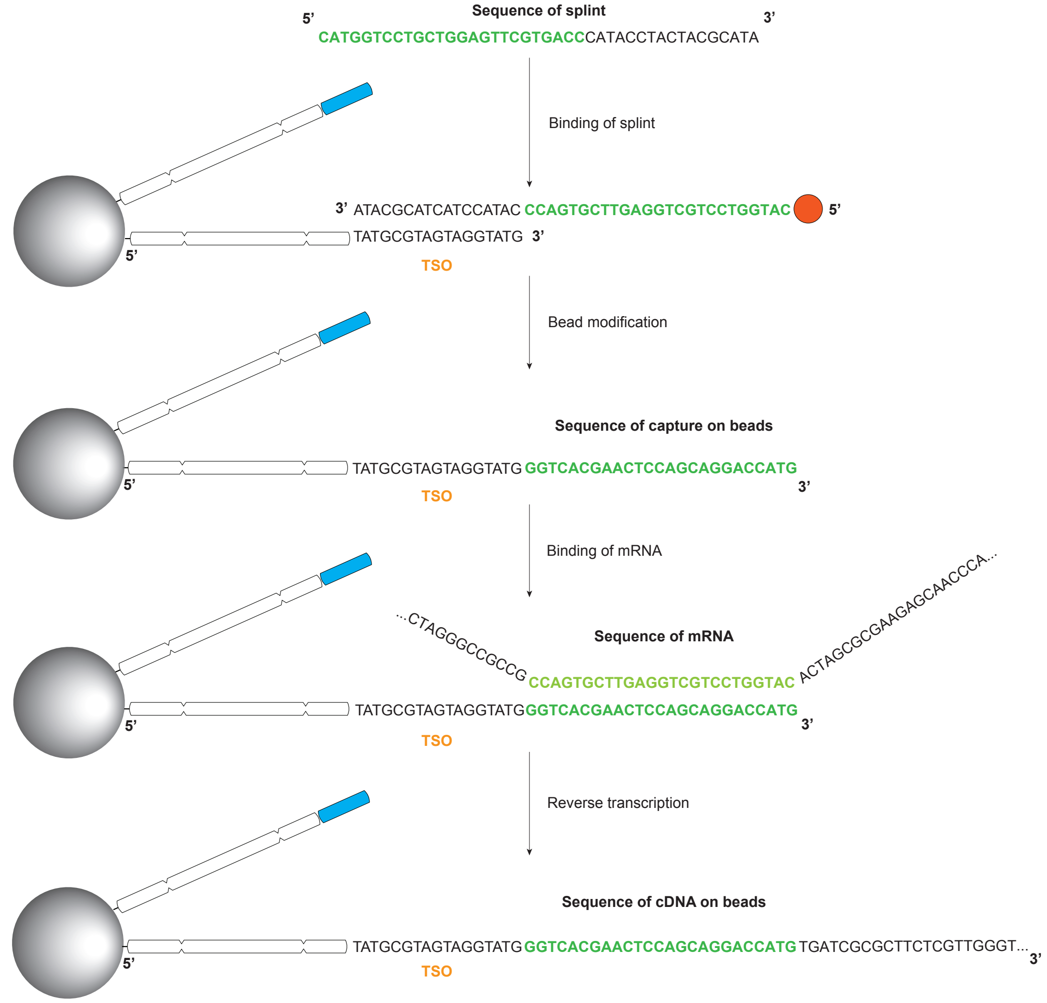

Supp Figure 2: Design of splints for RoCKseq bead modification

a, *eGFP* cDNA sequence. Grey: Untranslated Regions (UTRs), black: *eGFP* CDS, green: capture (RoCKseq) sequence. **b,** bead modification process to capture *eGFP*: splint binding, RoCKseq modification and target capture and reverse transcription. The splint contains three elements: a region complementary to the TSO sequence on the beads, the reverse complement of the capture sequence and a 5' phosphate group (red circle).

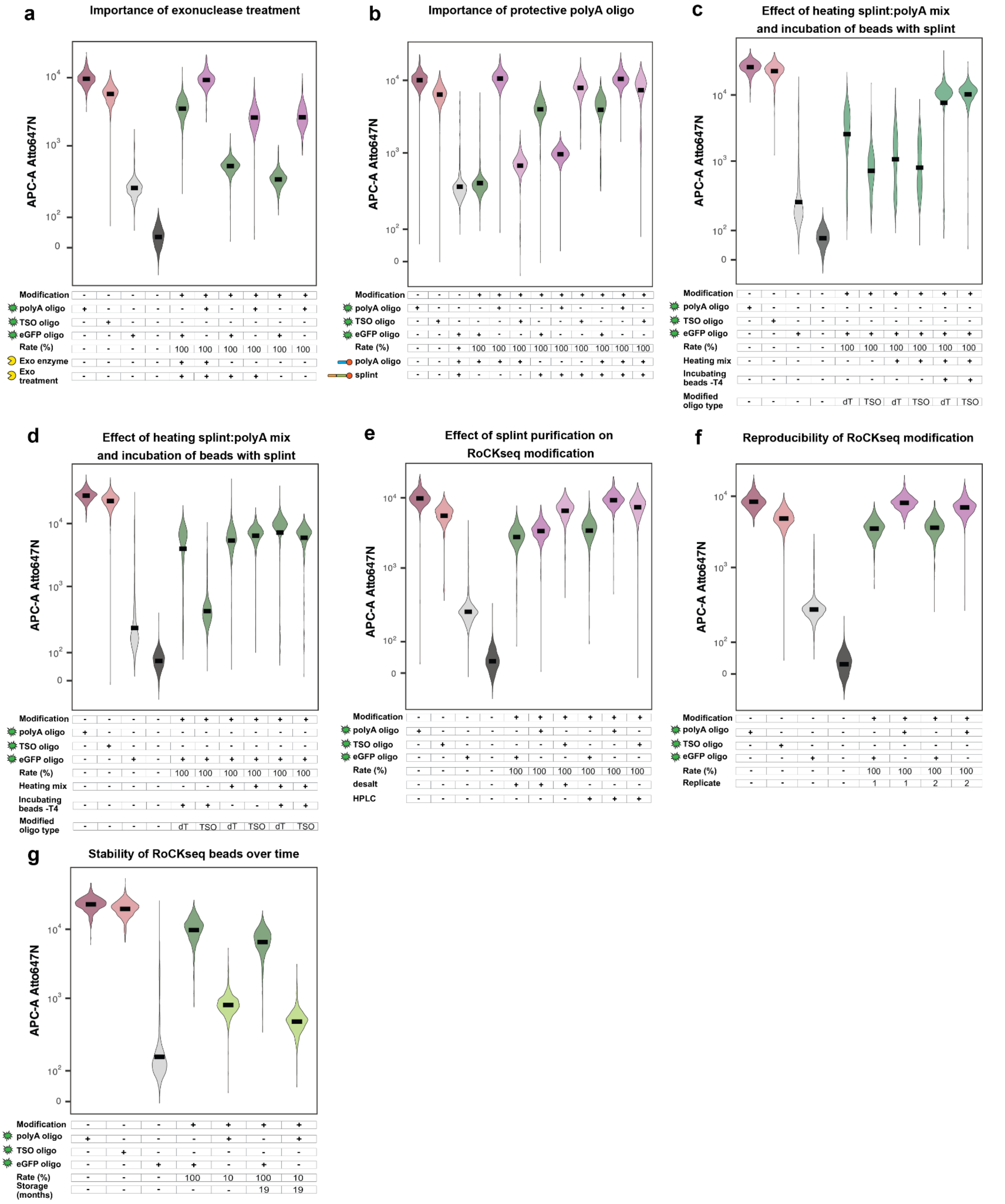

Supp Figure 3: Validation of RoCKseq bead modification

a-g, FACS quantification of RoCKseq bead modification. Effect of exonuclease treatment during RoCKseq bead modification **(a)**. Target: *eGFP* CDS. Exo enzyme: addition of exonuclease enzyme to reaction; Exo treatment: exonuclease step (including buffer and water, no enzyme). Effect of T4 polymerase 3' → 5' exonuclease activity on barcoded bead oligos **(b)**. Target: *eGFP* CDS. polyA oligo: only protective polyA oligo used for modification (omission of splint). splint: only splint used for modification (omission protective polyA oligo). Effect of heating of the splint/ polyA mix and incubation of beads with splint before addition of T4 polymerase enzyme **(c)**. Target: *eGFP* CDS. Heating mix: splint/ polyA mix was heated to 75°C for 5 minutes; Incubating beads -T4: beads were incubated with the splint at 37°C for 5

minutes before addition of the T4 polymerase. Modified oligo type: modification of dT or TSO oligos on BD Rhapsody beads. Effect of incubation of beads and splint before addition of T4 polymerase enzyme with or without heating of splint/ polyA mix **(d)**. Conditions as in **(c)**. Effect of purification level of splint and protective oligo on RoCKseq modification **(e)**. Target: *eGFP* CDS. desalt: RoCKseq bead modification with splint in desalted purification; HPLC: RoCKseq bead modification with splint with HPLC purification. Reproducibility of RoCKseq modification **(f)**. Target: *eGFP* CDS. Replicate: technical replicates of RoCKseq bead modification. Storage of RoCKseq beads **(g).** Target: *eGFP* CDS.

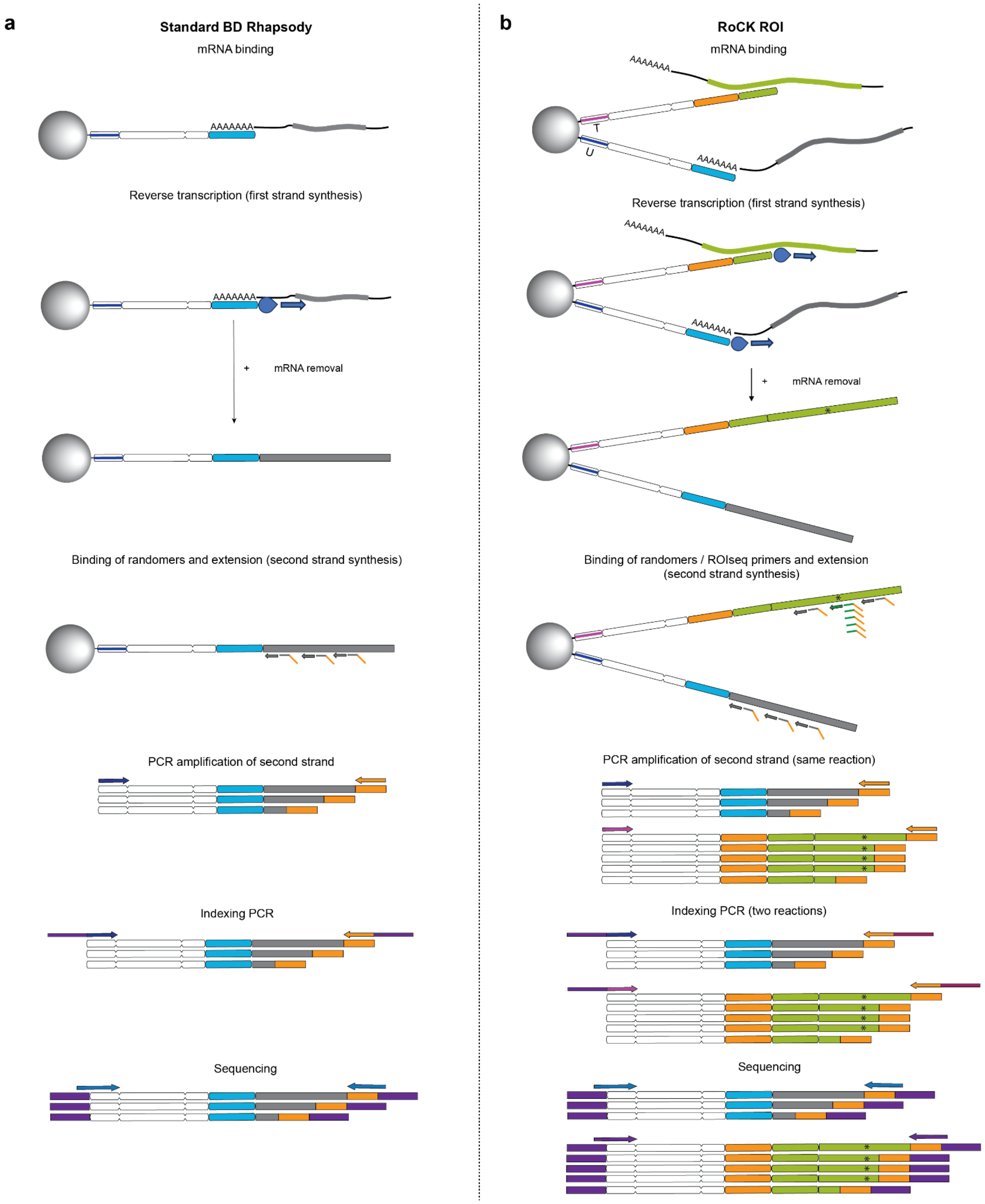

Supp Figure 4: Comparison of RoCK and ROI and standard BD Rhapsody library generation

a, Standard BD Rhapsody library generation. **b,** RoCK and ROI library generation using RoCKseq beads.

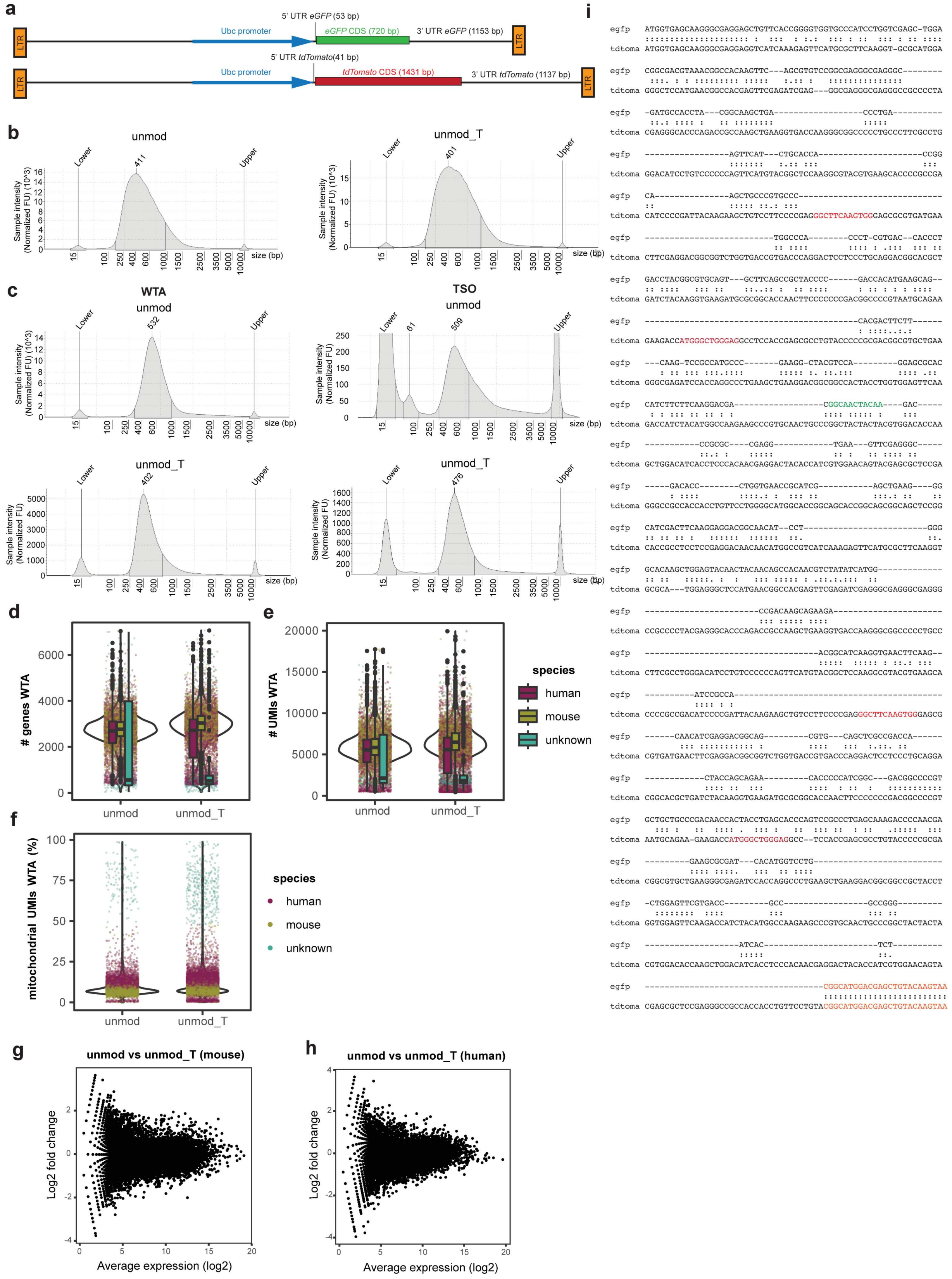

Supp Figure 5: Structure of transgenic construct in cell lines, quality control metrics for scRNA-seq experiment to test addition of T primer and position of RoCK and ROI primers in constructs

a, *eGFP* and *tdTomato* construct structure. 3' and 5' UTRs are identical (differences in number of bases are due to cloning). **b-c,** Library size distribution for unmod and unmod_T samples before **(b)** and after **(c)** indexing. **d-f,** Number of genes **(d)**, UMIs **(e)** and mitochondrial content **(f)**detected in WTA data on downsampled count tables. Figure legend in **(e)** applies to

(d) and **(e)**. **g-h**, MA plots for mouse cells **(g)** and human cells **(h)**. **i,** LALIGN local alignment of *eGFP* and *tdTomato* sequences showing the sequence similarities of the two CDSs**.** Orange: capture sequence used for the mixing experiments. Red: ROIseq primers for *tdTomato*. As *tdTomato* is a perfect repeat the ROIseq primers will bind twice. Green: ROIseq primer for *eGFP.*

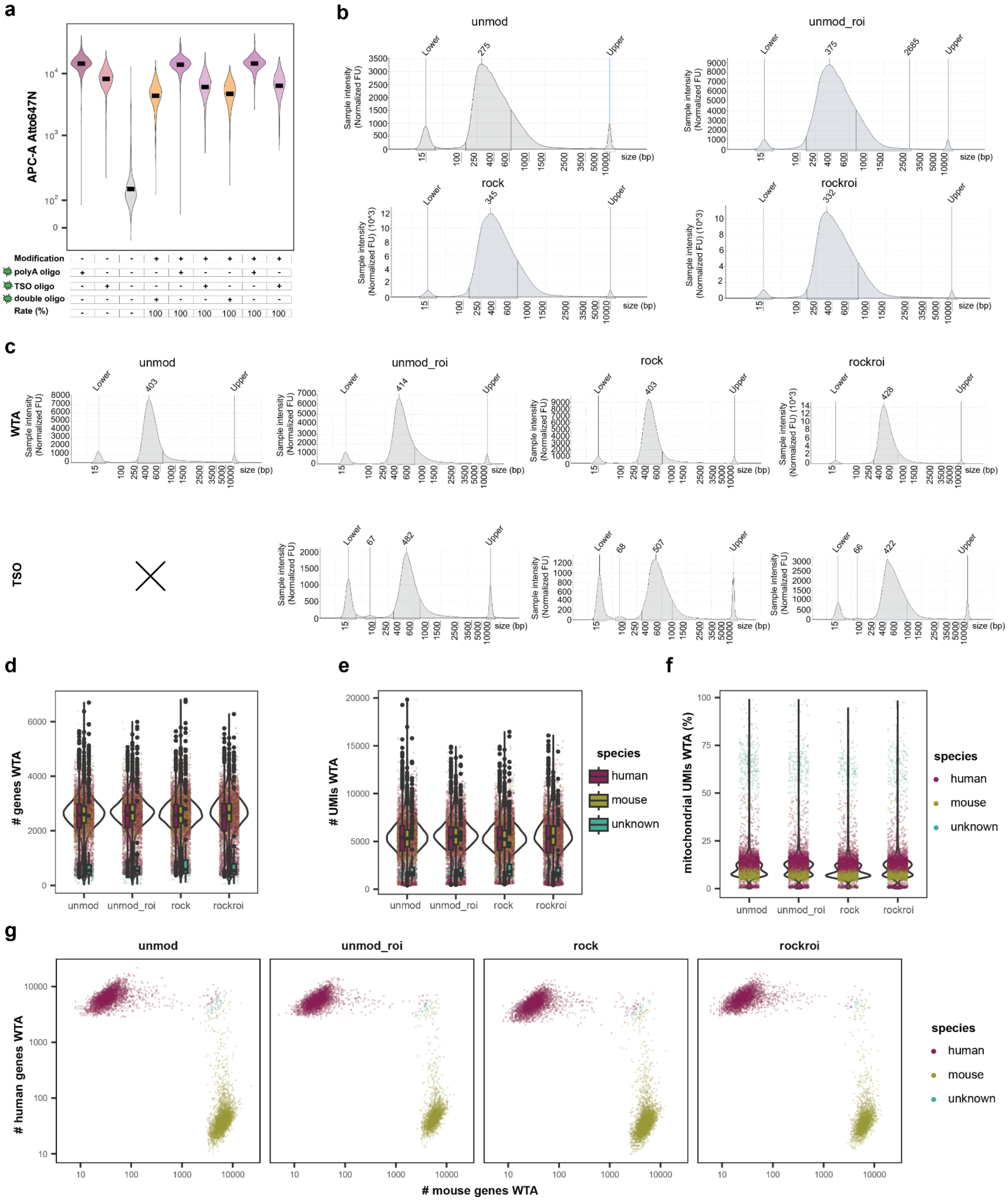

Supp Figure 6: Quality control metrics on WTA data for scRNA-seq experiment using mix of cell lines to test RoCK and ROI performance

a, FACS signal from modification of beads for scRNA-seq experiment**.** Y-axis: Atto647N fluorescent signal. The Y-axis has a biexponential transformation. **b-c,** Library sizes for unmod, unmod_roi, rock and rockroi samples before **(b)** and after **(c)** indexing. **d-f,** Number of genes **(d)**, UMIs **(e)** and mitochondrial content **(f)** detected in downsampled WTA data. Figure legend in **(e)** applies to **(d)** and **(e)**. **g**, Barnyard plot of species assignment using WTA data per condition.

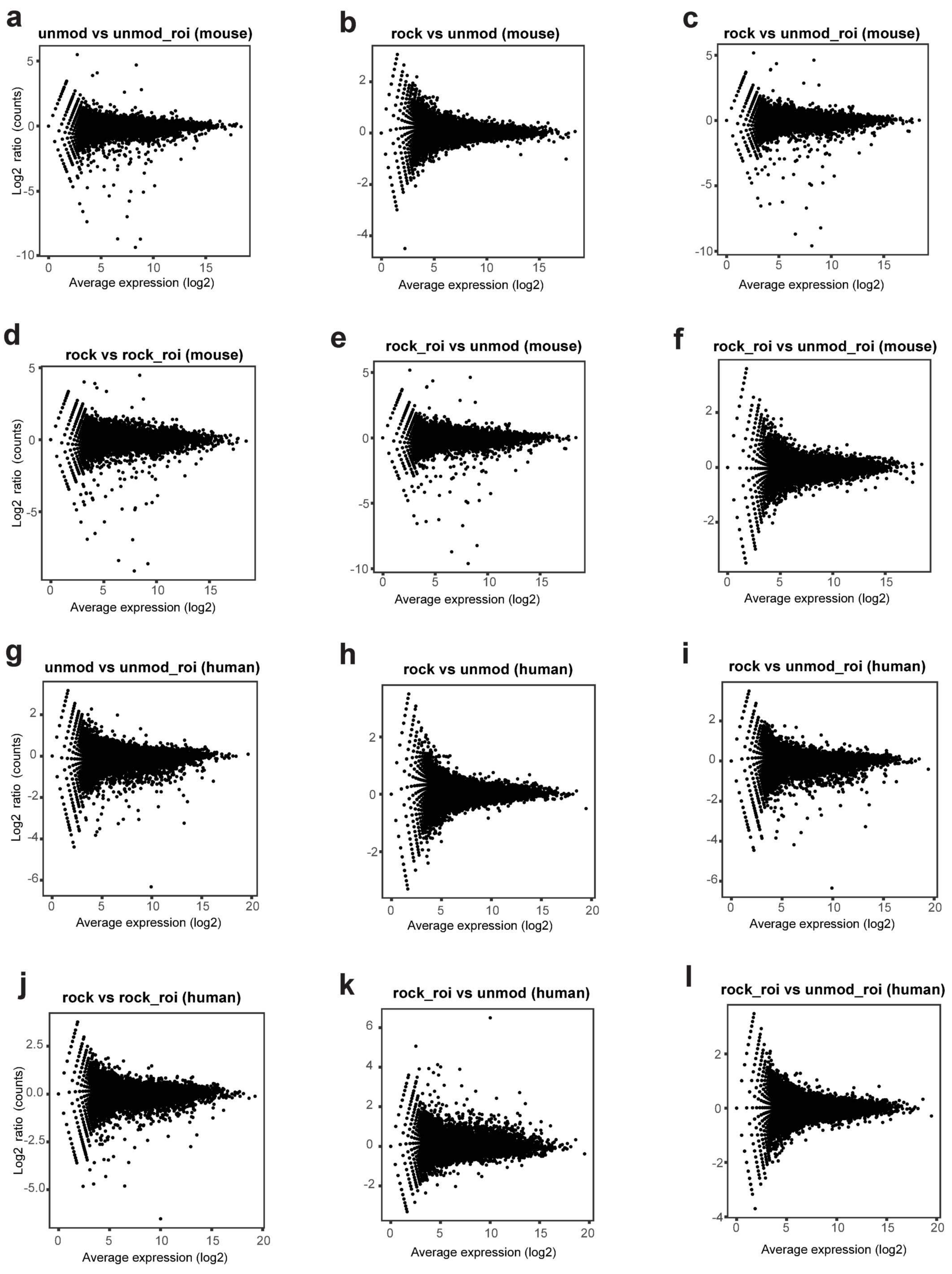

Supp Figure 7: Comparison of transcriptome for scRNA-seq experiment using mix of cell lines to test RoCK and ROI performance

a-l: MA plots for mouse cells (a-f) and human cells (g-l).

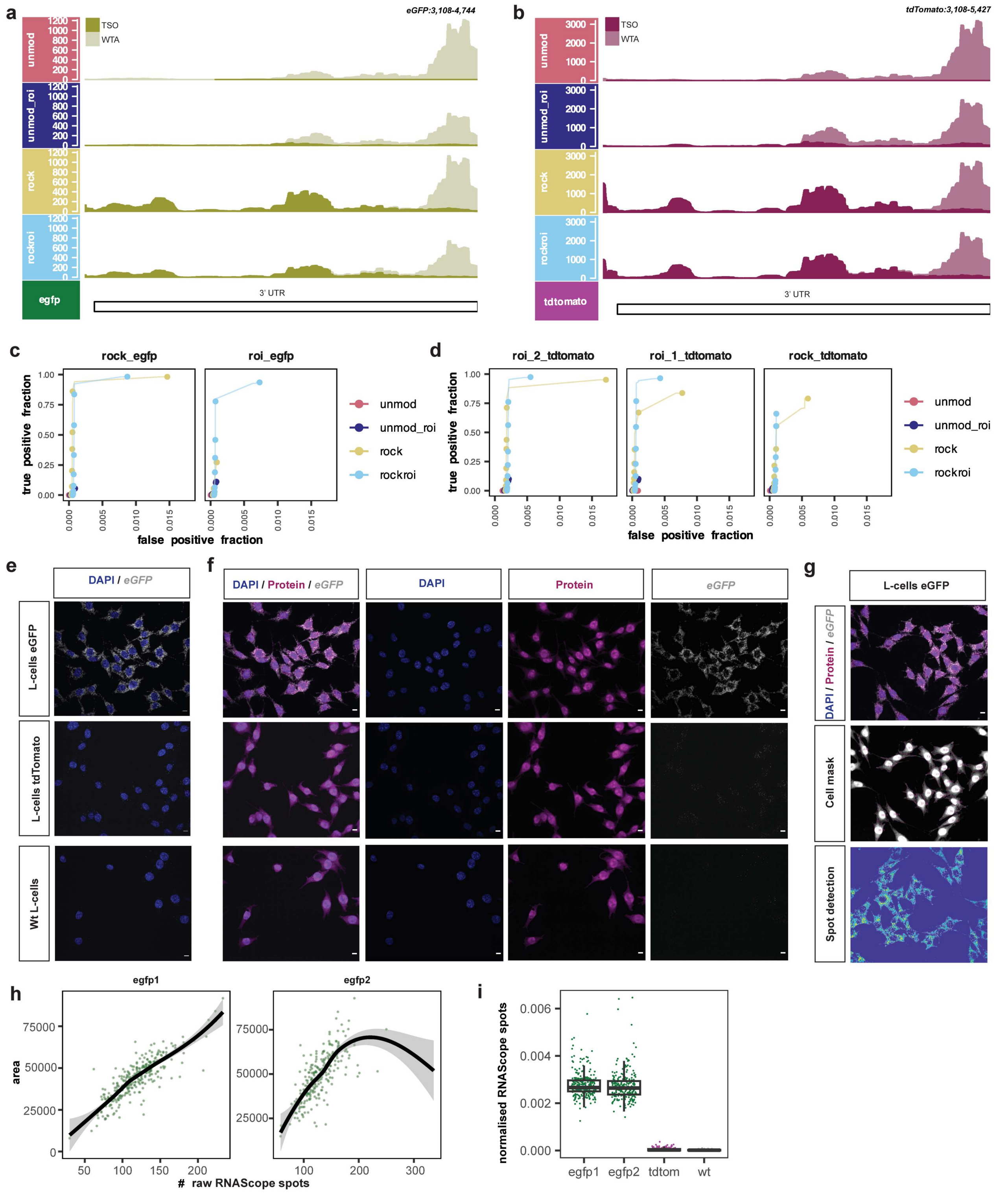

Supp Figure 8: Analysis of target data from scRNA-seq experiment using mix of cell lines to test RoCK and ROI performance and effect of cell area on quantification of *eGFP* mRNAs

a-b, Zoom in of 3' UTR from coverage plot in **Figure 4b (a)** and **Figure 4c (b).** The white bar indicates the zoomed-in 3' UTR. **c-d,** Receiver operating characteristic (ROC) curves indicating the true positive and false positive detection of RoCKseq and ROIseq regions for *eGFP* **(c)** and *tdTomato* **(d).** True positive fraction: detection of *eGFP* in mouse cells or *tdTomato* in human cells, respectively. False positive: detection of *eGFP* in human cells or *tdTomato* in mouse cells, respectively. **e-f,** Detection of *eGFP* transcript in L-cells expressing eGFP, tdTomato or wt L-cells (untransduced) without **(e)** or with **(f)** protein stain. Scale bars: 10 µm. **g,** Example of cell mask and spot detection on L-cells expressing eGFP. Scale bars: 10 µm. h, Number of RNAScope spots versus cell area for the two replicates (egfp1 and egfp2). Source data

are provided as a Source Data file. i, Number of RNAScope spots normalized by cell area. Source data are provided as a Source Data file. Boxplots show the median (center line), the first and third quartiles (bounds of the box; 25th and 75th percentiles), the lower and upper whiskers (1.5 × IQR from the box).

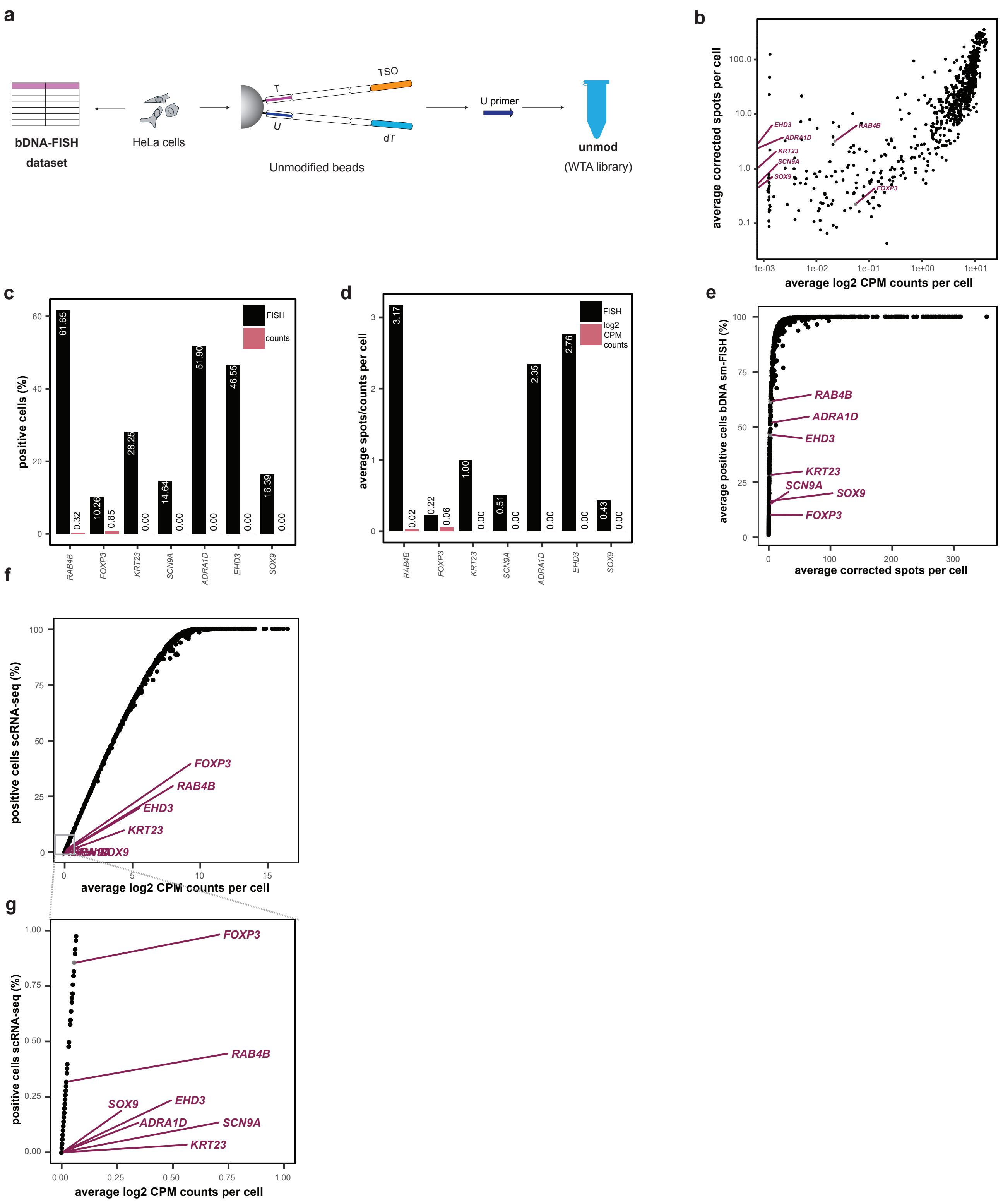

Supp Figure 9: Analysis of unmodified condition performed on HeLa cells

a, Experimental set up of unmodified condition. Primers: U. **b**, Average log2 CPM counts per cell per gene (each dot represents a gene) versus average corrected spots per cell. Genes which were later targeted in the modified experiment are indicated. **c**, Percent of positive cells with detectable bDNA sm-FISH or counts. **d**, Average bDNA sm-FISH spots or log2 CPM counts per cell. **e**, Average corrected spots per cell versus average positive cells detected in bDNA sm-FISH. Genes which were later targeted in the modified experiment are indicated. **f**, Average log2 CPM counts per cell per gene (each dot represents a gene) versus average positive cells detected in scRNA-seq. Genes which were later targeted in the

modified experiment are indicated. **g)** Zoom in of panel **(f)**. Average corrected spots per cell (**b-e**) calculated on genes detected in both bDNA sm-FISH replicates***.***

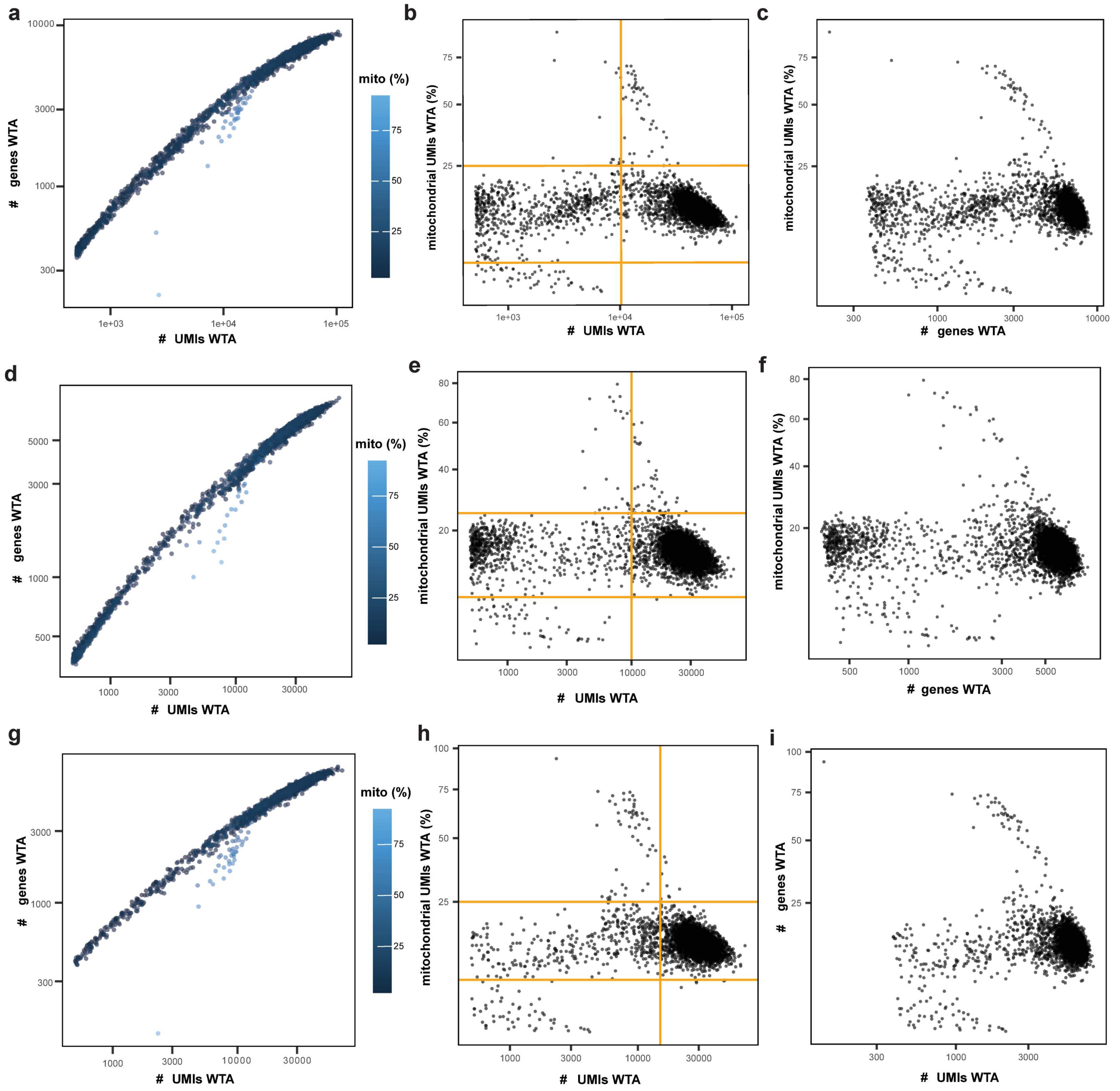

Supp Figure 10: Quality control on unmodified and modified conditions performed on HeLa cells

a, Number of genes versus number of UMIs colored by mitochondrial content, unmodified sample. **b**, Mitochondrial content versus number of UMIs, unmodified sample. **c**, Mitochondrial content versus number of genes, unmodified sample. **d**, Number of genes versus number of UMIs colored by mitochondrial content, modified sample. **e**, Mitochondrial content versus number of UMIs, modified sample. **f**, Number of genes versus number of UMIs colored by mitochondrial content, modified sample. **g**, Number of genes versus number of UMIs colored by mitochondrial content, downsampled unmodified sample. **h**, Mitochondrial content versus number of UMIs, downsampled unmodified sample. **i**, Mitochondrial content versus number of genes, downsampled unmodified sample. Orange lines: QC filtering thresholds.

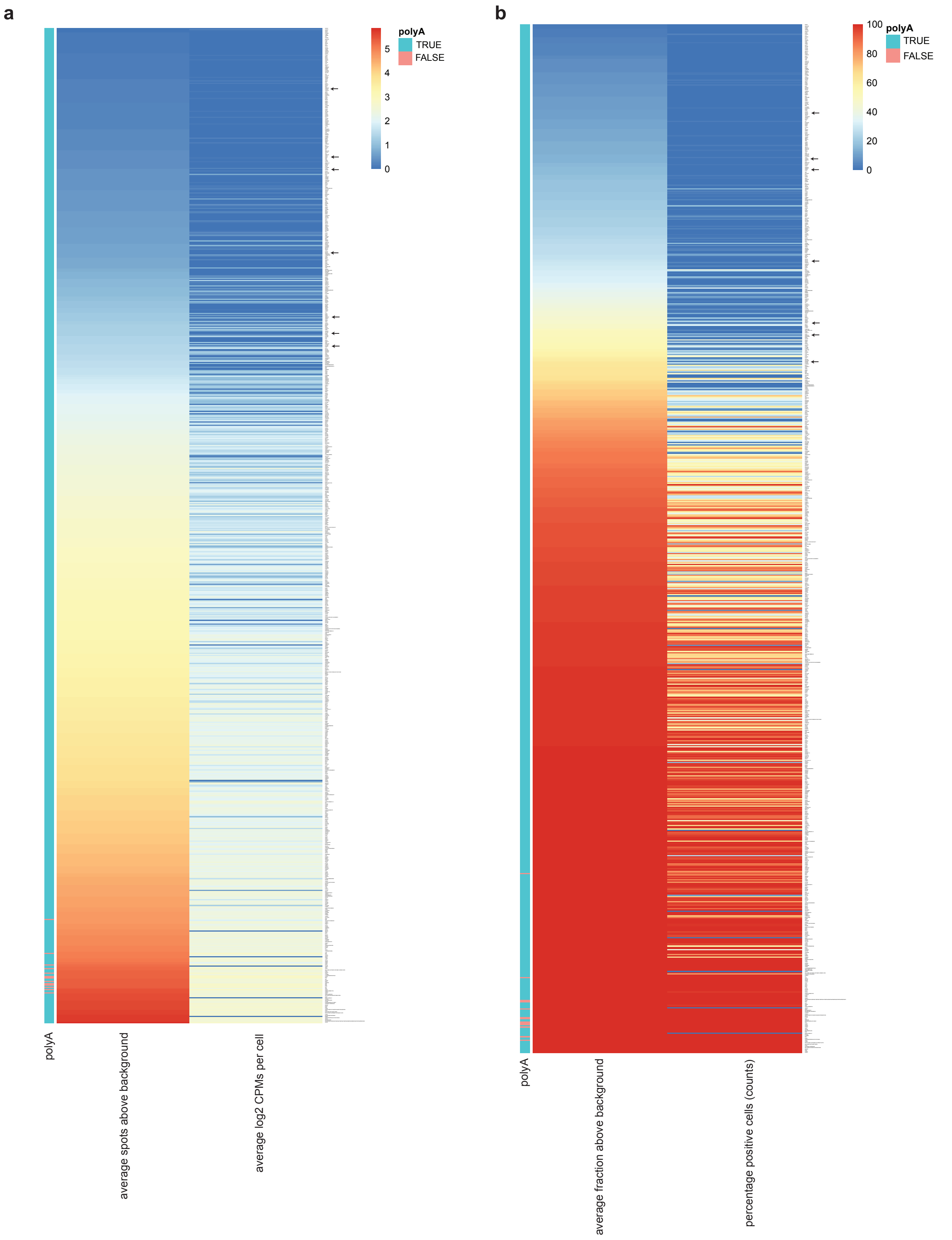

Supp Figure 11: Heatmaps on transcript detection in scRNA-seq compared to bDNA sm-FISH

a, Average spots above background (bDNA sm-FISH) compared to average log2 CPMs per cell. Average corrected spots per cell calculated on genes detected in both bDNA-FISH replicates. **b**, Average fraction above background (bDNA sm-FISH) compared to percentage of positive cells (counts)

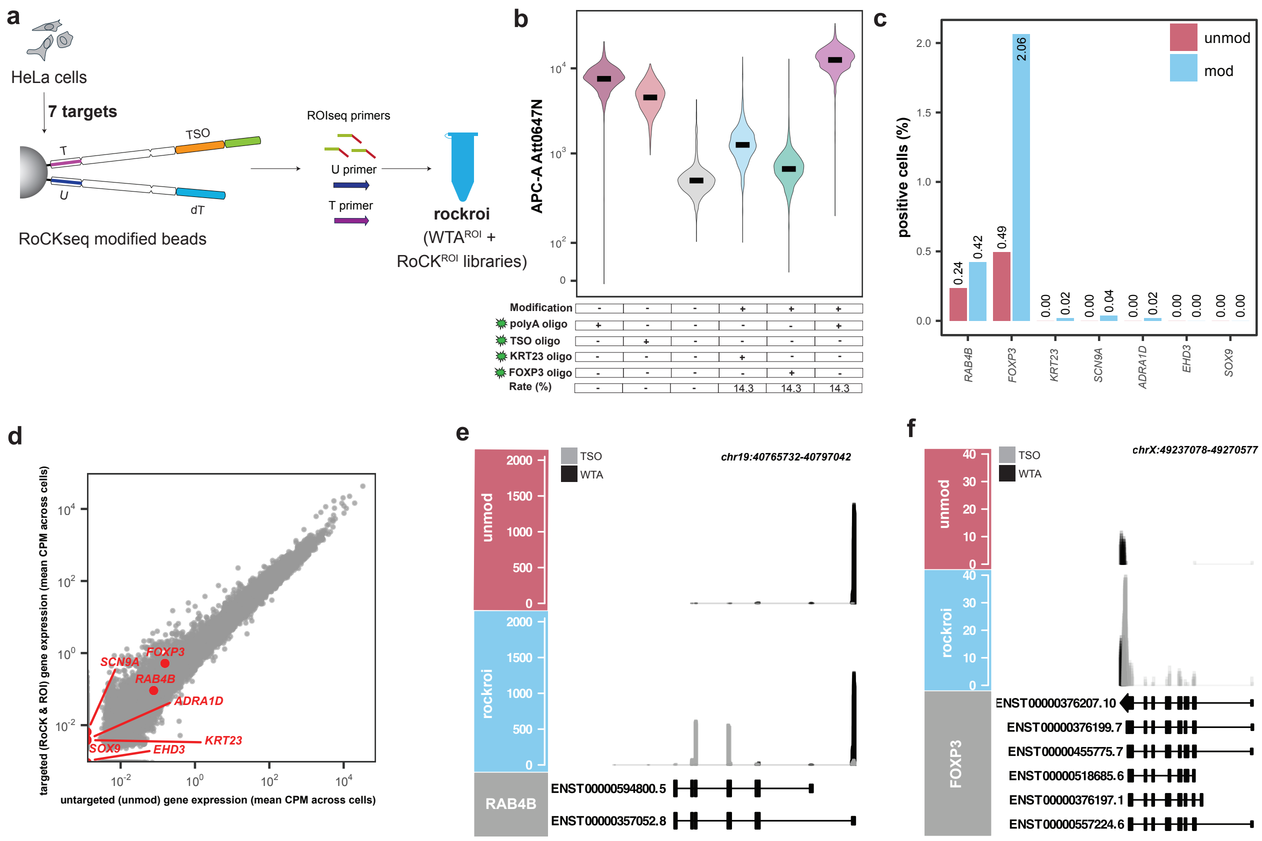

Supp Figure 12: Analysis of modified experiment performed on HeLa cells

a, Experimental set up of unmodified condition. Primers: U and T. **b**, FACS signal from modification of beads for scRNA--seq experiment. Y-axis: Atto647N fluorescent signal. The y axis has a biexponential transformation. **c**, Percent of positive cells for the seven targets in the modified compared to the unmodified sample. **d)** Mean CPMs across cells for unmodified versus modified sample. Red dot indicates the targeted transcripts. Unique alignments were considered for all genes. **e-f**, Sequence coverage for TSO (grey) and WTA (black) along **(e)** RAB4B, **(f)** FOXP3. Plots **(c-f)**: plots were made using a downsampled unmodified data (see Methods)

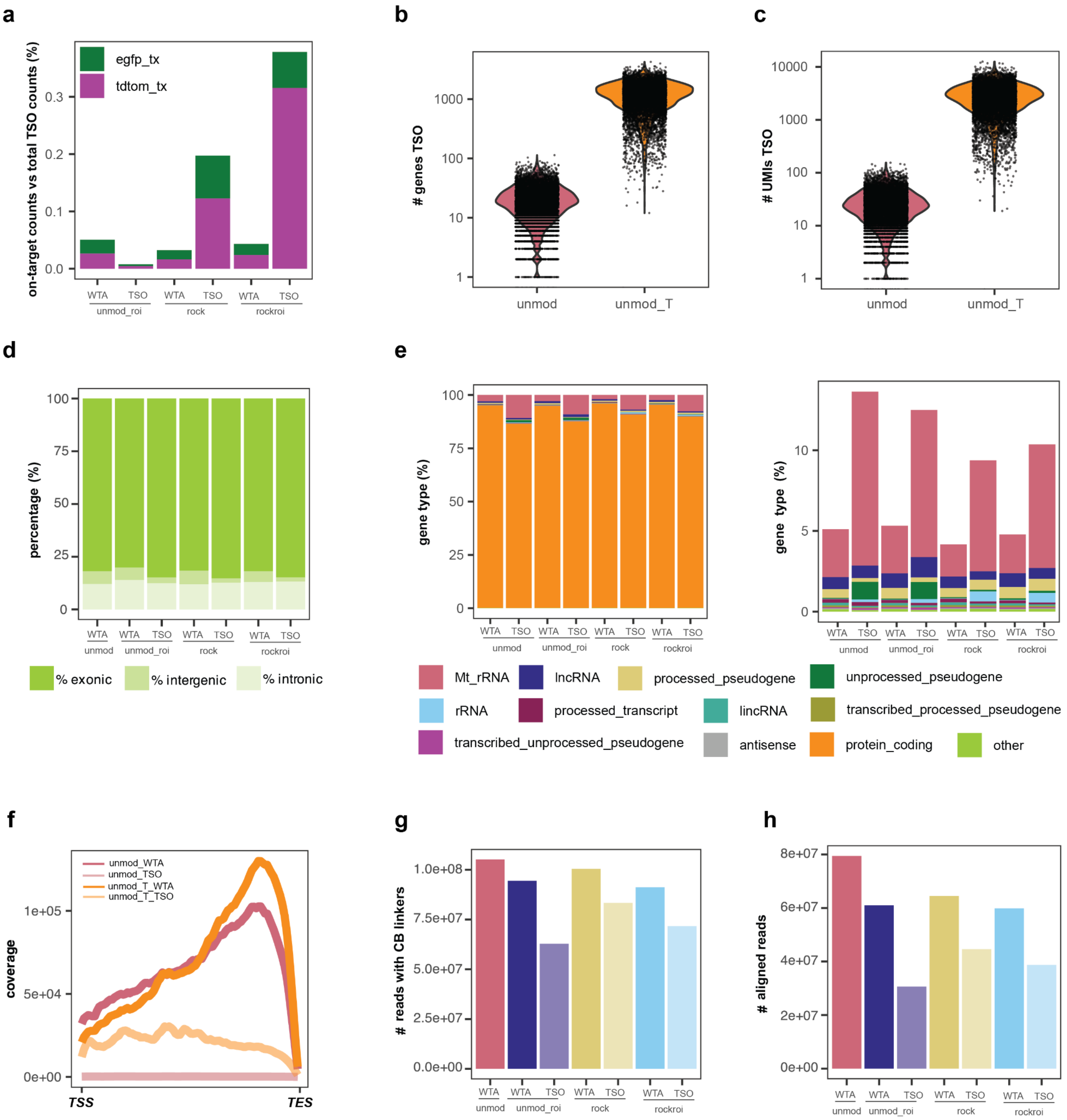

Supp Figure 13: Characterization of TSO data from RoCK and ROI experiments

a, On-target counts versus total TSO counts for *eGFP* and *tdTomato* across conditions, including CDS and UTRs. **b-c,** Number of genes **(b)** and UMIs **(c)** detected in the TSO data**. d,** Percent exonic, intergenic and intronic alignments in WTA and TSO data. **e,** Top 11 gene biotypes detected in TSO and WTA data with (right) and without (left) protein coding genes **(e).** Other: all other gene types not in the top 11. **f,** Aggregated gene body sequencing coverage along all transcripts detected in TSO and WTA TSS: transcription start site, TES: transcription end site. **g,** Number of raw reads with canonical WTA and TSO cell barcodes, regardless of whitelists. **h,** Number of aligned reads **(h)**. Numbers indicate the total number of alignments. Data in panels **(a, d-e, g-h)** refer to experiment described in **Figure 3 (a)**, data in panels **(b-c, f)** refer to experiment described in **Figure 2 (b)**.

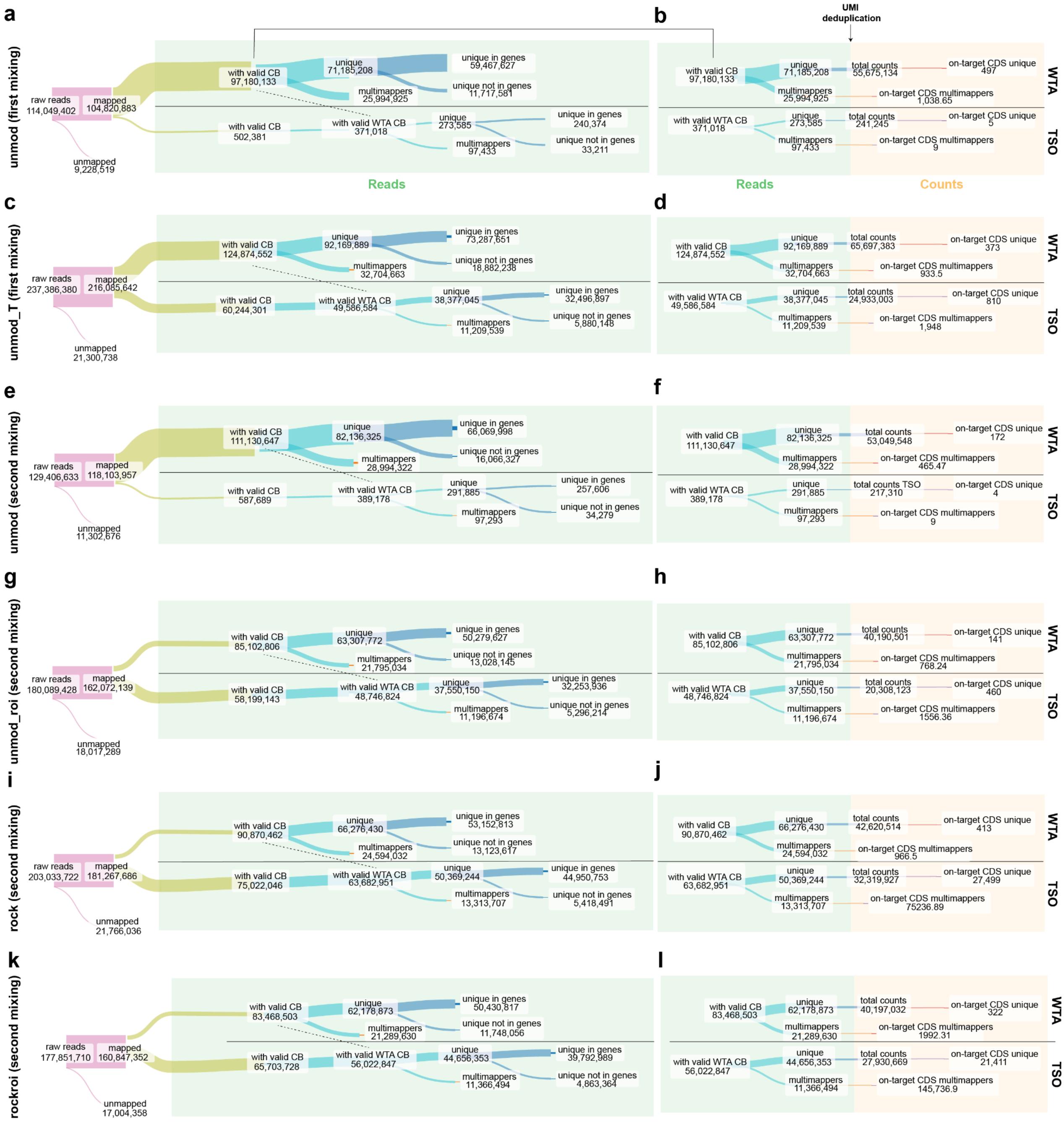

Supp Figure 14: Flow of reads during RoCK and ROI scRNA-seq human and mouse mixing experiments

a-l, Sankey plots depicting the WTA and TSO sequencing, alignment, UMI deduplication and cell barcode detection performance across conditions (mouse and human mixing experiments)**.** Conditions: unmod (first mixing) and unmod_T (first mixing) for experiment described in **Figure 2 (b) (panels a-d)**; unmod (second mixing), unmod_roi (second mixing), rock (second mixing) and rockroi (second mixing) for experiment described in **Figure 3 (a) (panels e-l)**. Dashed line: filtering of TSO reads with non-empty cells with valid cell barcodes as detected in WTA data. Nodes: raw reads: number of reads from FASTQ files; mapped and unmapped: reads mapped to genome or not; with valid CB (WTA): WTA reads after EmptyDrop-filtering of cell barcodes (CB) from empty wells; with valid CB (TSO): TSO reads with a valid cell barcode structure; with valid WTA CB (TSO): TSO reads with cell barcodes matching WTA's EmptyDrops-filtered cells; unique and multimappers: uniquely and multimapping reads, respectively; unique in genes and unique not in genes: uniquely mapped reads overlapping genes or outside genes, respectively; total counts: total number of (gene) counts after UMI deduplication; on-target CDS unique and on-target CDS multimappers: number of *eGFP* and *tdTomato* counts in their CDS according to the multimapping status of the original read. Counts are 1/n transformed, n being the number of compatible loci (n=1 for unique reads). CDS: coding region.

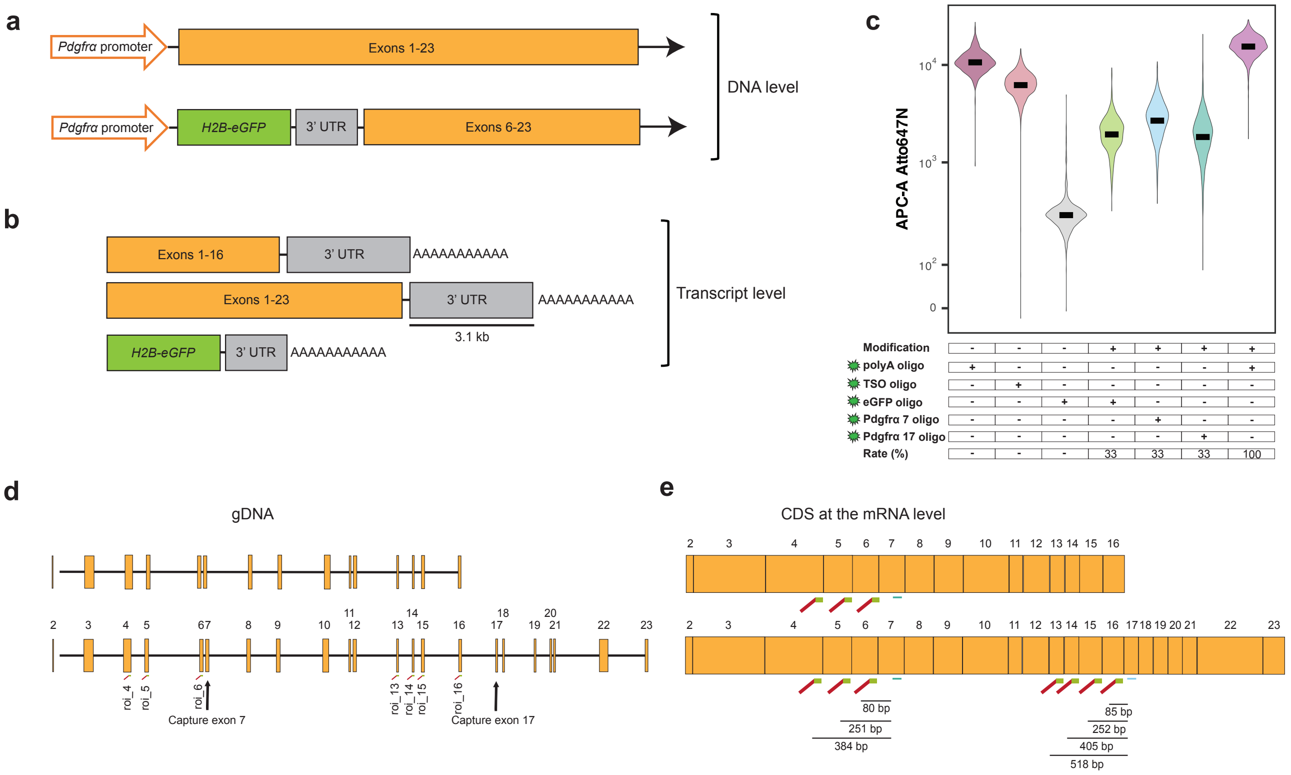

Supp Figure 15: *Pdgfra* locus, capture, regions of interest and bead modification

a, Structure of the *Pdgfrα* locus in the transgenic mouse strain used for the scRNA-seq experiment**.** The mouse strain harbors a *Pdgfrα* allele where the first four exons were substituted with an *H2B-eGFP* construct. **b,** Structure of the *Pdgfrα*-derived transcripts from **(a).** Top two diagrams: long and short *Pdgfrα* isoforms, last diagram: transcript derived from *H2B-eGFP* transgenic allele. **c,** FACS signal from modification of beads for scRNA-seq experiment**.** Y-axis: Atto647N fluorescent signal. The Y-axis has a biexponential transformation. **d-e,** Exons targeted via RoCKseq captures and ROIseq primers for the short (top, ENSMUST00000202681.3 and ENSMUST00000201711.3) and long (bottom, ENSMUST00000000476.14 and ENSMUST00000168162.4) *Pdgfrα* isoforms at the gDNA **(d)** and mRNA **(e)** levels**.**

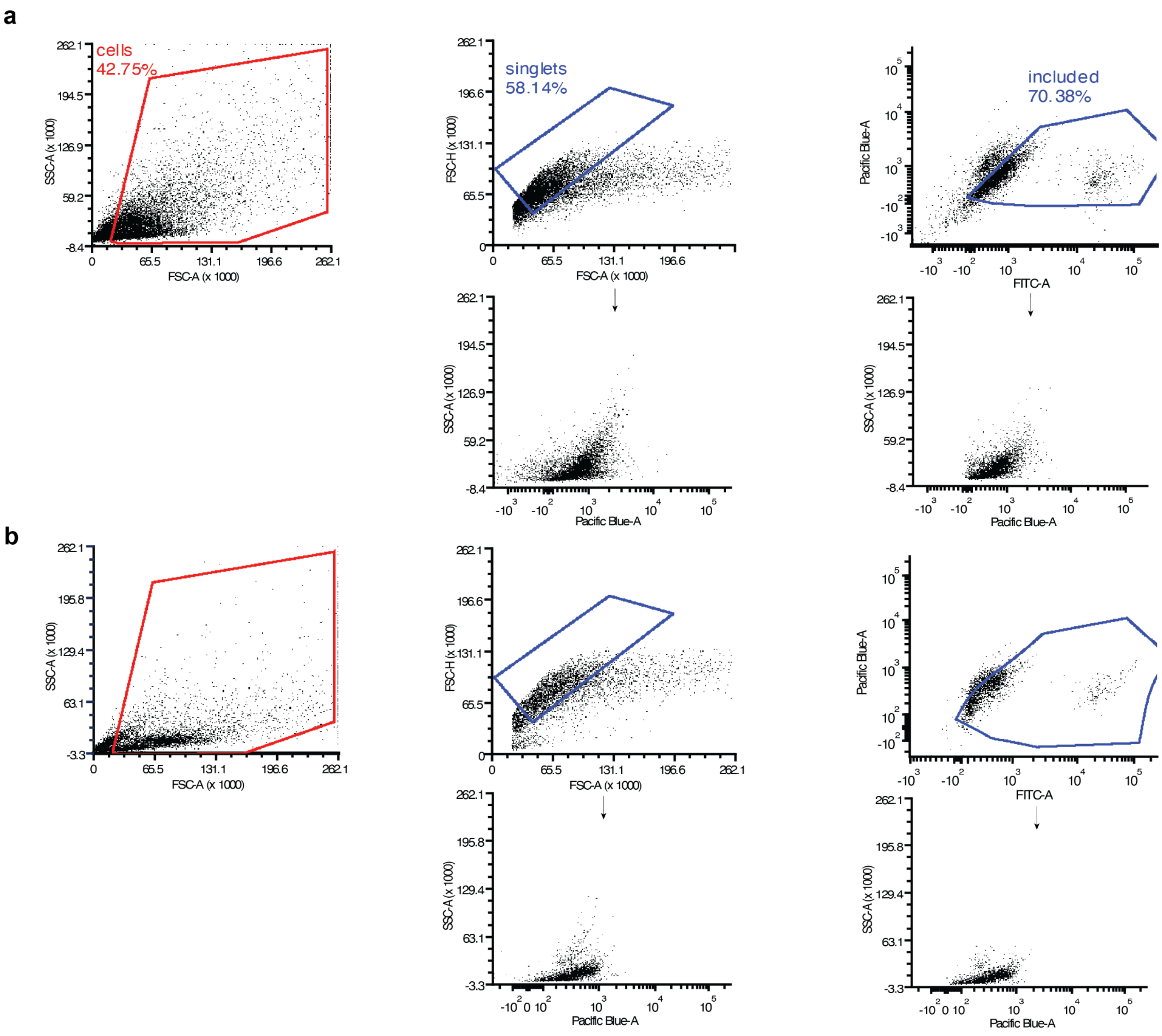

Supp Figure 16: Cell gating for murine colonic cells

a, FACS gating of the mesenchymal fraction. Gating of cells was done on forward scatter (FSC-A) versus side scatter (SSC-A) signal, while singlets were gated in FSC-A versus FSC-H signal. Live cells (included gate) were gated on FITC-A (signal from eGFP positive cells) versus Pacific Blue-A (viability signal). Bottom: additional plots (Pacific Blue-A for live cells versus SSC-A) showing gating for live cells (left: gated for singlets, right: gated for included). **b,** FACS analysis of negative control without viability staining**.** Gating of cells was done on FSC-A versus SCC-A signal, while singlets were gated in FSC-A versus FSC-H signal. Arrows connect plots that have the same gating but are represented in different channels.

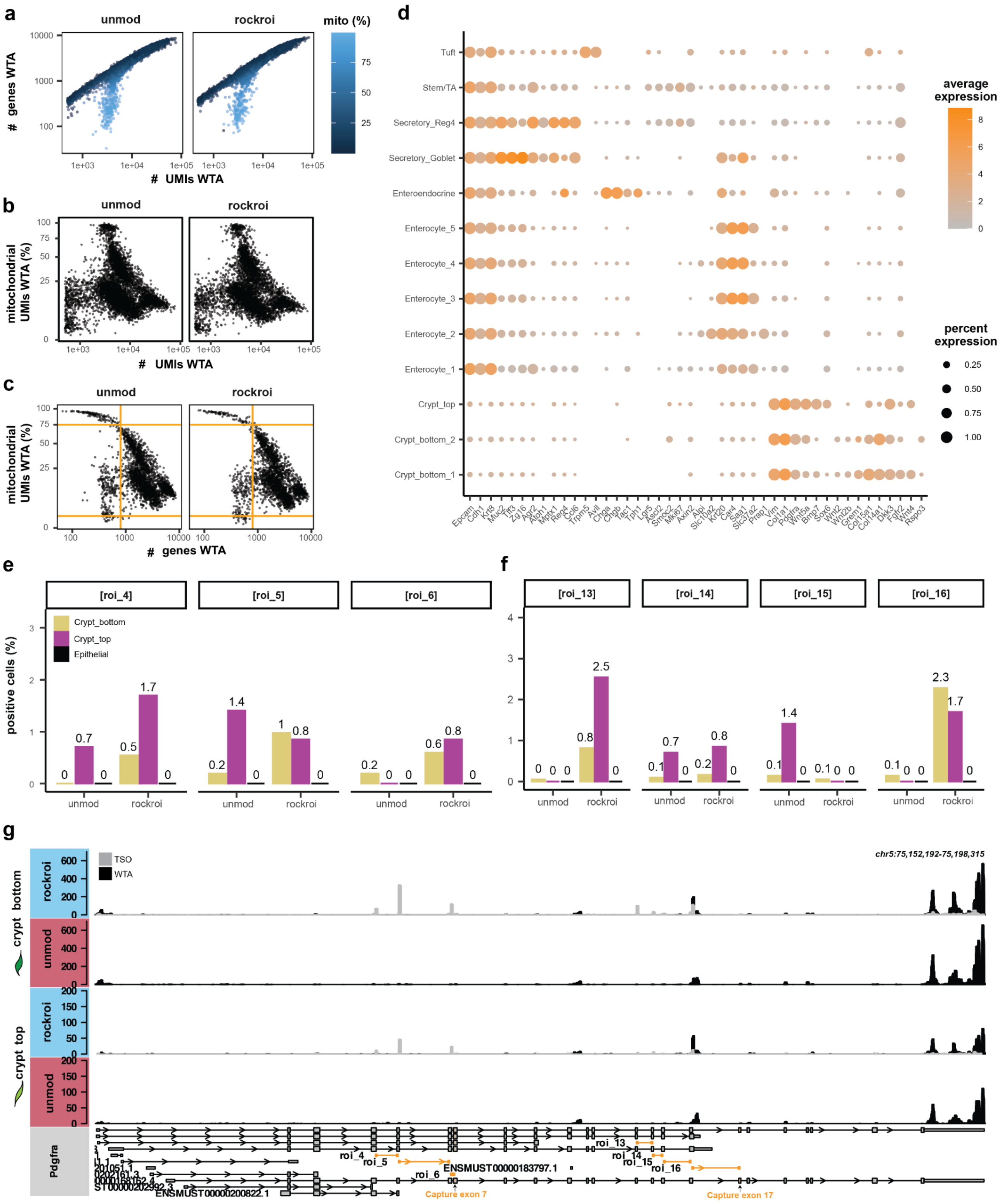

Supp Figure 17: Quality control of *Pdgfra* scRNA-seq experiment, descriptive analysis and splicing quantification **a,** Number of genes versus number of UMIs colored by mitochondrial content, downsampled WTA data. **b,** Mitochondrial content versus number of UMIs, downsampled WTA data**. c,** Mitochondrial content versus number of genes**.** Orange lines: quality control (QC) filtering thresholds. **d,** Expression of manual cell type annotation markers across cell clusters**. e-f,** Percent positive cells in which at least one UMI spanning the splice junction targeted by ROIseq was detected on WTA data. **g,** Coverage along *Pdgfra* split by crypt top and crypt bottom fibroblasts for TSO (gray) and WTA (black) libraries.

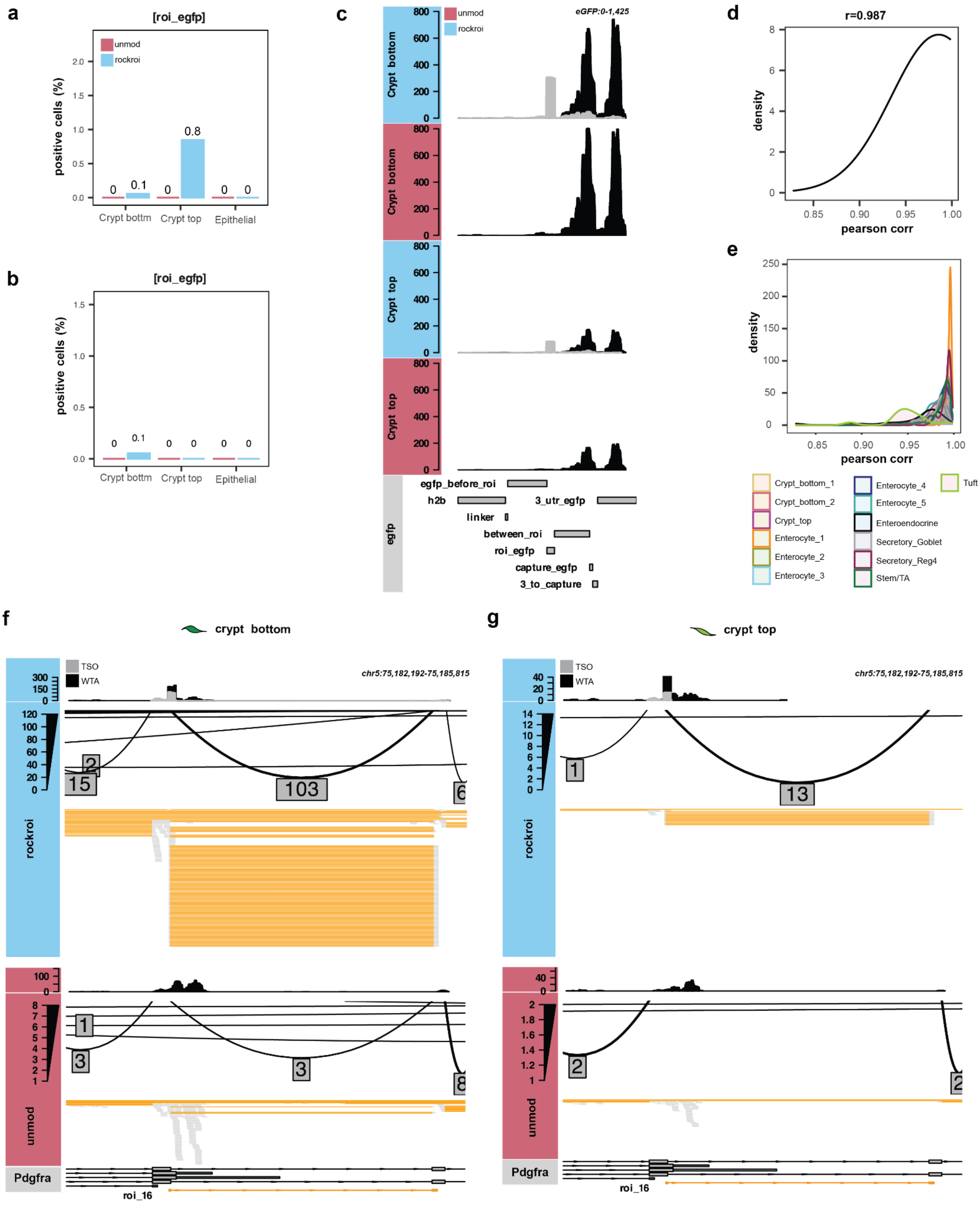

Supp Figure 18: Detection of *Pdgfra* alternative splicing

a-b, Percent positive cells in which at least one UMI for the ROI^{eGFP} (labelled as roi_egfp in plot) was detected on TSO **(a)** or WTA data **(b)**. **c,** Coverage along e*GFP*. **d-e,** Pearson correlation distributions between same barcodes in the unimodal and multimodal rockroi WTA data for all cells **(d)** or split by cell type (**e**). Correlations were calculated on 100 genes**. f-g,** Coverage, sashimi and alignment tracks for roi_16 region in crypt bottom **(f)** or crypt top **(g)** fibroblasts**.** Boxed values indicate the number of alignments spanning splice junctions.

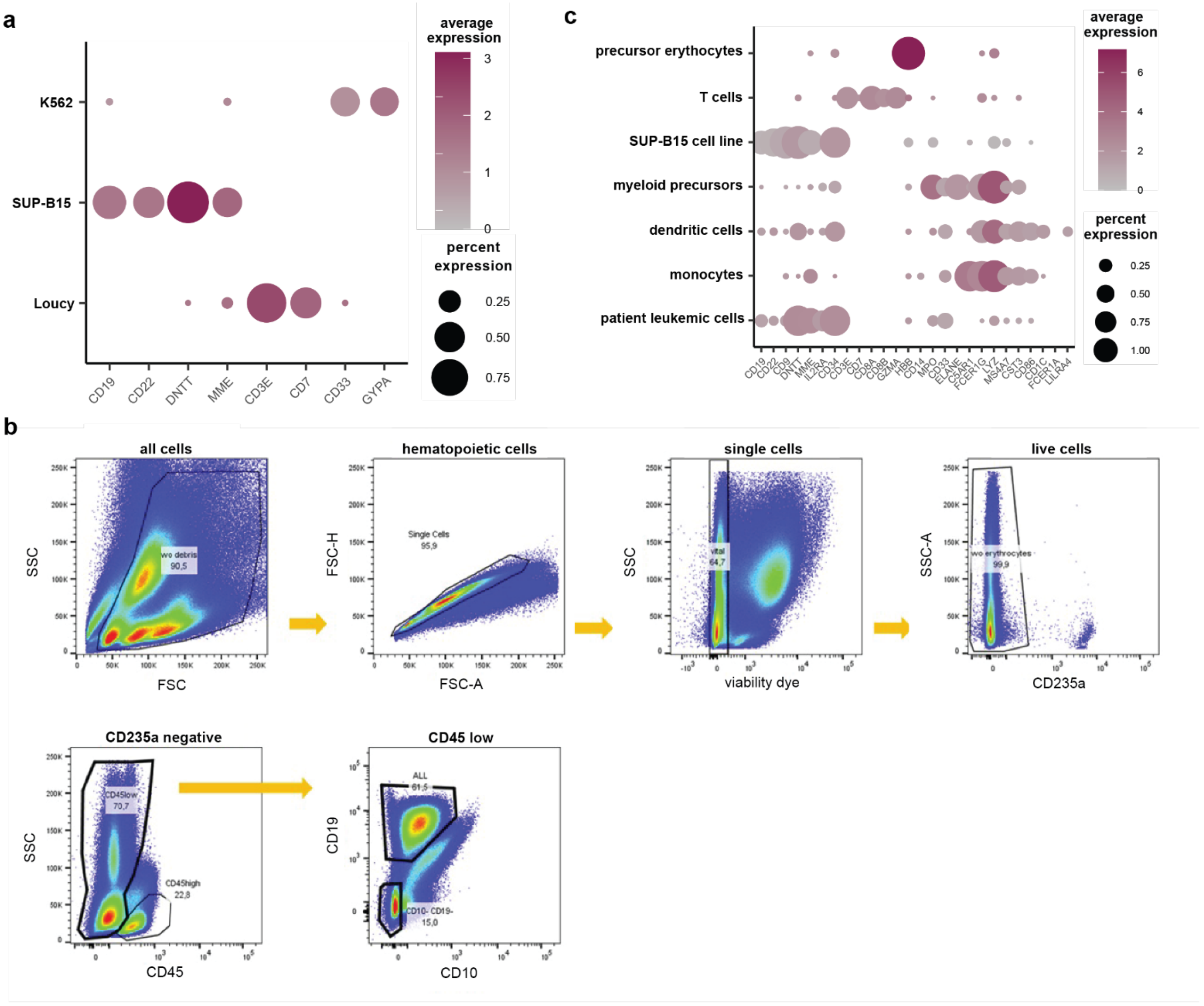

Supp Figure 19: Detection of the *BCR::ABL1* fusion transcript in leukemia cells

a, Expression of manual cell type annotation markers used for annotation of the cell line Uniform Manifold Approximation and Projection (UMAP) across cell clusters**. b,** Sorting strategy of patient sample for enrichment of non-leukemic compartment. Flow cytometry scheme used to sort non-leukemic compartment (CD235a-CD45lowCD19-CD10-) and B cell precursor cells/leukemic cells (CD235a-CD45lowCD19+) from human frozen bone marrow at primary diagnosis of BCP-ALL. **c**, Expression of manual cell type annotation markers used for annotation of patient and cell line UMAP across cell clusters**.**

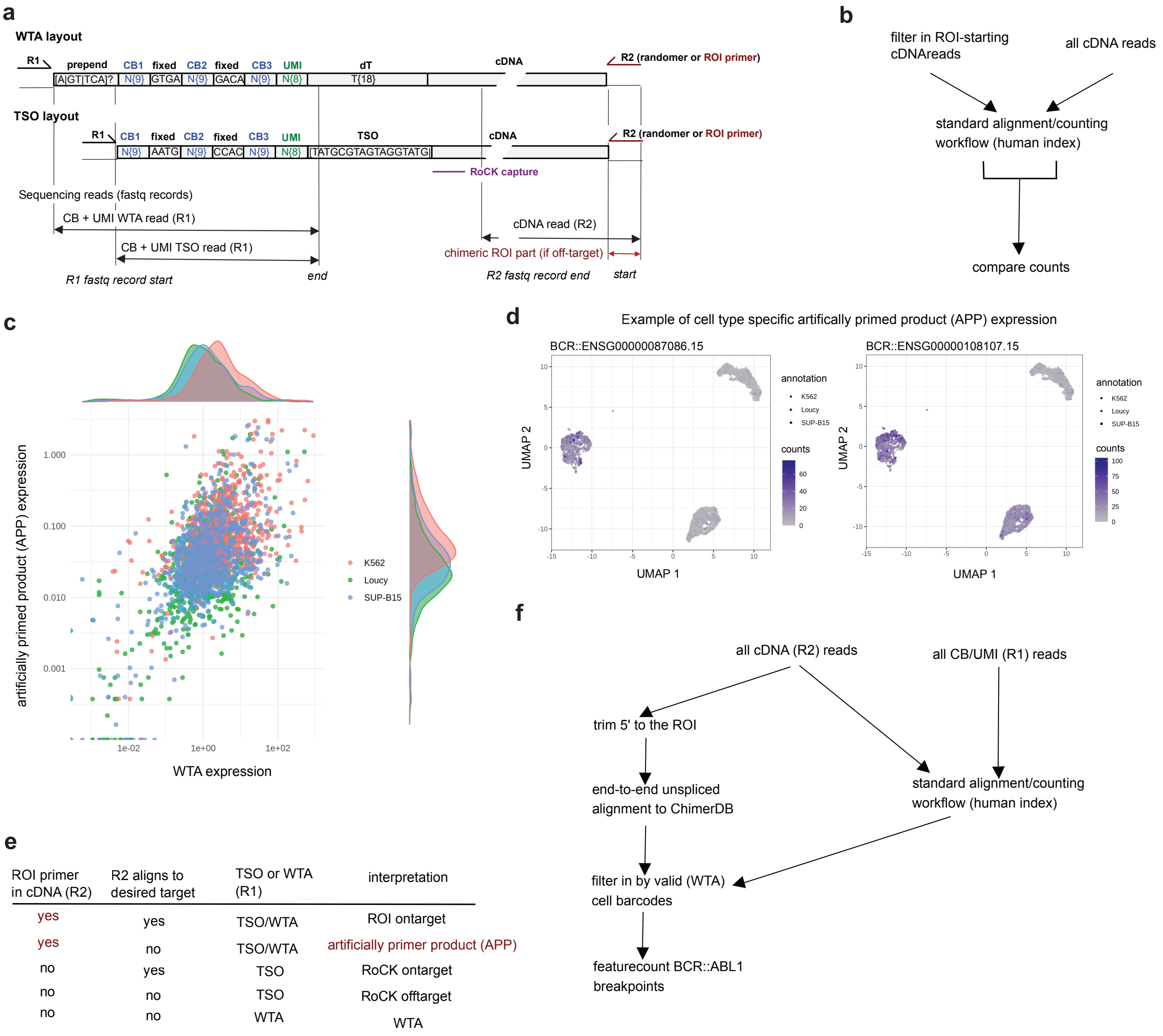

Supp Figure 20: Analysis of artificially primed products (APPs)

a, BD Rhapsody tripartite cell barcode (CB, blue), UMI (green; Unique Molecular Identifiers) and cDNA layout, including RoCK (capture) and ROI (priming) locations, and potential APP cDNA read artifacts (chimeric ROI^{BCR} sequence in red). **b**, Exploratory analysis of APPs abundance. **c**, *BCR*-containing APPs are pervasive using standard alignment **(b)**, with read counts depending on the untargeted (WTA) gene expression level. Each dot depicts a ROI^{BCR}::gene APP (e.g., for any gene, ABL1 or not) detection from a (leukemia) cell line. **d,** APPs can be cell line-specific, as showcased by ROI^{BCR}::FTL (left) and ROI^{BCR}::RPL278 (right) using the standard (b) quantification. e, Summary of the defining characteristics of RoCK and ROI off-targets and APPs. f, Custom workflow to quantify end-to-end *BCR::ABL1* true gene fusions.

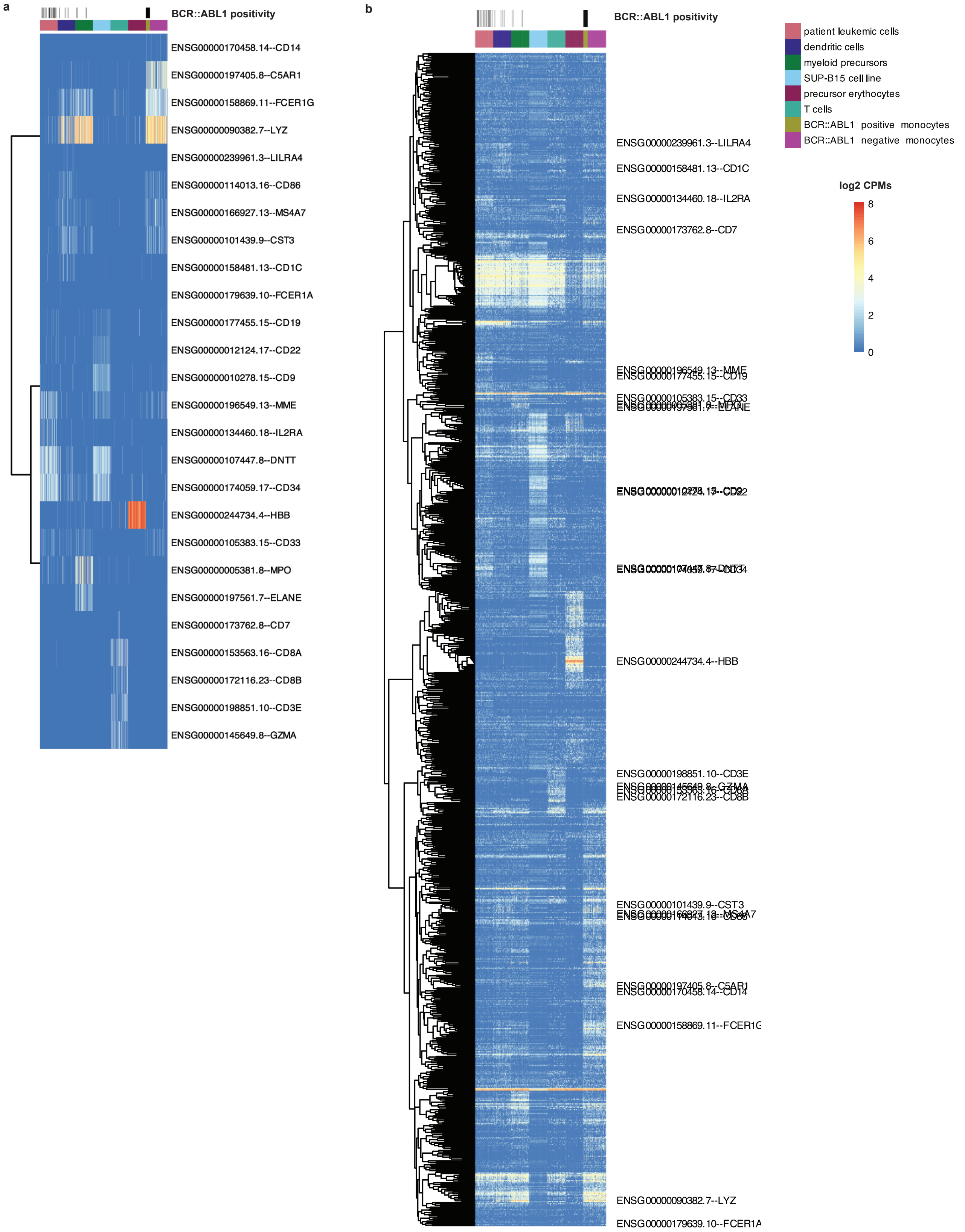

Supp Figure 21: Comparison of *BCR::ABL1* positive and negative monocytes

a, Heatmap of cell types and markers used for annotation, with monocytes split between *BCR::ABL1*-positive and negative; **b,** Heatmap of cell types and markers used for annotation including top 1’000 highly variable genes, with monocytes split between *BCR::ABL1*-positive and negative.

Supplementary Tables

Supp Table 1: scRNA-seq experiments with relevant metrics including sequencing depth and number of cells before and after filtering. Table is also provided as Supplementary Data 1 for higher resolution.

Supp Table 2: Comparison of additional time needed for targeting and maximal number of cells which can be recovered from single scRNA-seq run.

Supp Table 3: Calculations to obtain read depth per cell.

Supp Table 4: Calculations to obtain cost per cell.

Supp Table 5: Additional targeting costs for ROCK and ROI scRNA-seq experiments.

Supp Table 6: Additional targeting costs for DART-seq scRNA-seq experiments.

Supp Table 7: Sensitivity of targeting.

Supp Table 8: Number of targets for each scRNA-seq method.

Supp Table 9: Detection of off-targets in scRNA-seq experiments.

Supp Table 10: Markers used for annotation of murine colonic cell clusters.

Supp Table 11: Primer sequences. Table is also provided as Supplementary Data 2 for higher resolution.

Supp Table 1: scRNA-seq experiments with relevant metrics including sequencing depth and number of cells before and after filtering.

name_experiment	name_fastqs (R[1,2])	SRR_identifier	conditions	modalities	bead_modification	umber_of_ROIseq_prime	number_of_reads_per_sample	mber_of_cells_per_condition_(unfilte	r_of_cells_per_condition_(after_QC_filtering_and_doublet_removal)
First cell line mixing experiment	o307161_1-Unmodified_S4_R*_001.fastq.gz	SRR28817193	unmod	WTA	No	0	114049402	9768	8587
First cell line mixing experiment	o307161_2-Unmodified_N1_S1_R*_001.fastq.gz	SRR28817192	unmod_T	WTA / TSO	No	0	237386380	10091	7966
Second cell line mixing experiment	315641_1-Unmod_S4_R*_001.fastq.gz	SRR28830618	unmod	WTA	No	0	129406633	8020	6775
Second cell line mixing experiment	315641_2-Unmod_ROI_S2_R*_001.fastq.gz	SRR28830617	unmod_roi	WTA / TSO	No	3	180089428	7003	5724
Second cell line mixing experiment	315641_3-RoCK_S1_R*_001.fastq.gz	SRR28830616	rock	WTA / TSO	Yes	0	203033722	8226	7118
Second cell line mixing experiment	315641_4-RoCK_ROI_S3_R*_001.fastq.gz	SRR28830615	rockroi	WTA / TSO	Yes	3	177851710	6476	5231
Pdgfra epithelial mesenchymal cells	325411_1-Unmod_WTA_S1_R*_001.fastq.gz	SRR28839349	unmod	WTA	No	0	379866683	5711	4634
Pdgfra epithelial mesenchymal cells	325411_2-RoCK_ROI_WTA_S2_R*_001.fastq.gz	SRR28839348	rockroi_unimodal	WTA	Yes	8	368399364	4980	4275
Pdgfra epithelial mesenchymal cells	325402_1-RoCK_ROI_WTA_S1_R*_001.fastq.gz	SRR28839350	rockroi_multimodal	WTA / TSO	Yes	8	665200527	5079	4097
BCR::ABL1 fusion cell lines	331131_1-Cell_lines_50_50_S1_R*_001.fastq.gz	NA	rockroi	WTA / TSO	Yes	2	465285612	6101	5434
BCR::ABL1 fusion patient sample	331131_2-Patient_S2_R*_001.fastq.gz	NA	rockroi	WTA / TSO	Yes	2	383759722	6455	5675
HeLa cells unmodified	384021_1-Unmod_1_S1_R*_001.fastq.gz	SRR35038871	unmod	WTA	No	0	1109565519	6114	5034
HeLa cells modified	387722_1-RoCK_ROI_7_S2_R*_001.fastq.gz	SRR35038870	rockroi	WTA / TSO	Yes	7	1139901915	6307	5429

Supp Table 2: Comparison of additional time needed for targeting and maximal number of cells which can be recovered from single scRNA-seq run.

Times are calculated based on protocol described in original publication. **NA:** no available.

	Standard protocol	Additional targeting time	Maximal number cells
**RoCK and ROI**	BD Rhapsody	2 hours	40’000
**DART-seq** [2]	DROP-seq	2 hours	20’000
**HyPR-seq**[3]	Custom	Day 1: 2 h, overnight, day 2: 8h	30’000
**TARGET-seq**[4]	Custom	total: 23 h	384
**BART-seq**[5]	Custom	Day 1: 6 h, overnight, day 2: 4 h 30 min	3’000
**GoT-seq**[6]	10x	30 mins (GoT-seq)<br>2 x overnight, day 2/ 3; 6h (Circ GoTseq)	10’000
**GoT-Splice**[7]	10x	6 h	10’000
**Chigene**[8]	Custom	NA	15’000
**BD Targeted Amplification**[9]	BD Rhapsody	3 h 30 mins	40’000
**RAGE-seq**[10]	10x	Day 1: 2 h, 2x overnight, day 2/3: 4 h	10’000
**Van Horebeek et al.**[11]	10x	1 h 30 mins	10’000
**Constellation-seq**[12]	DROP-seq<br>10x	40 mins (DROP-seq)<br>2 h (10x)	10’000
**scTalLoR-seq**[13]	10x	Day 1: 2 h, overnight, day 2: 3 h	10’000
**scTLR-seq**[14]	10x	3 h 30 mins	10’000
**scCapture-seq**[15]	Multiple	3 days incubation + 2 h	384

Supp Table 3: Calculations to obtain read depth per cell.
# reads per experiment: M: millions. average reported for separate experiments if values for each experiment differ largely. *# cells*: number of recovered cells after sequencing. *Exp*: experiment. *ONT:* Oxford Nanopore Technology. *^a*: value reported by study. If not specified value extrapolated from deposited data. *NA:* values not reported in table, either because data not available or because the read depth per cell was directly reported in the study. *R1:* read 1, *R2:* read 2. Reads needed to obtain information on index are not reported. ***:** values are based on averages reported in Dataset S02 (excluding information on 10x libraries). ** values are based on the pluripotency experiment (GSE130422)**.** *** values are based on P1 and P2 experiments for targeted experiments (Table 1).

	# reads / experiment (M)	Sequencing machine	Read set up	# cells	Read depth cell
**RoCK and ROI**	272	Illumina NovaSeq 6000^	R1=60, R2=62	7’500	36’000
**DART-seq** [2]	Exp 1: 30<br>Exp 2: 680	Illumina NextSeq 500^b	R1=20, R2=130-131	1’000^b	Exp 1: 30’00<br>Exp 2: 680’000
**HyPR-seq**[3]**,***	50	Illumina NextSeq 550^b	R1=40-43, R2=12	2’532^b	19’747
**TARGET-seq**[4]	NA	Illumina NextSeq 500/550^b	Full-length: R1=75, R2=75^b<br>3’ - biased: R1=28, R2=64^b	Full-length: 458^b<br>3’ - biased: 2’798^b	Full-length: 2.4 M^b<br>3’ - biased: 152,552 reads^b
**BART-seq**[5]**,****	23.5	Illumina Miseq^b	R1=128, R2=132	4’500^b	5’200
**GoT-seq**[6]	GoT: 40<br>Circularization GoT: 0.35	GoT: Illumina HiSeq 2500 / Illumina MiSeq^b<br>Circularization GoT: Illumina MiSeq^b<br>Long-read: Nanopore GridION X5^b	GoT: R1=26/28, R2=98/130^b<br>Circularization GoT: R1=15 R2=150^b	10’000^b	GoT: 4’000<br>Circularizatio GoT: 35
**GoT-Splice**[7]	NA	Nanopore PromethION GridION / MinION^b	Long-read	10’000^b	NA
**Chigene**[8]	NA	MGI-T7^b	R1=150, R2=150^b	NA	NA
**BD Targeted Amplification**[9]	40	Illumina HiSeq 2500^b	R1=150, R2=150^b	8’000^b	2,165 / 14,042^b
**RAGE-seq**[10]	ONT: 20<br>10x: 312	ONT: Nanopore MIN106^b<br>10x: Illumina NextSeq 500^b	10x: R1=150, R2=150^b	5’000^b	ONT: 4’000<br>10x: 60’000^
**Van Horebeek et al.**[11]**,***	0.5 M^b (based on P1 an P2)	Illumina NextSeq500 / Illumina MiSeq^b	R1=28, R2=150^b	3’000^b	170
**Constellation-seq**[1]	NA	Illumina Nextseq500^b	10x: R1= 28, R2=60^b<br>DROPseq: R1=20, R2=50^	10x: 1’000^b<br>DROPseq: 6’000^b	10x: 1’500^b<br>DROPseq: 20’000^b
**scTalLoR-seq**[13]	Exp 1: 70<br>Exp 2: 340	Exp 1: Nanopore MinION^b, Exp 2: Nanopore PromethION^	Long-read	10’000^b	Exp 1: 7’000<br>Exp 2: 34’00
**scTLR-seq**[14]	NA	Nanopore GridION	Long-read	10’000^b	NA
**scCapture-seq**[15]	1 per cell^b	Illumina HiSeq 4000	R1=75, R2=75^b	1’000^b	1’000’000

Supp Table 4: Calculations to obtain cost per cell.

Cost per cell is calculated on values reported by studies. The cost for RoCK and ROI and DART-seq experiments were calculated in detail (Supp Table 4 and 5). *Sequencing cost:* cost needed to sequence libraries to a similar read depth reported in Supp Table 2. Costs are based on values from the FGCZ sequencing facility in Zurich, Switzerland. *# cells*: number of cells used for calculations. Numbers are based on maximal amount of cells which can be loaded. *Standard library cost*: cost to generate standard library. The cost is based on Zhang, et al., 2019[16] and Gao et al., 2020[17] and does not include the instrument cost. Costs are not calculated for custom platforms. *Targeting cost*: additional cost which is needed for targeting step of protocol. ^*a:* value reported by paper**.** *NA:* data not available. * Sequencing costs for RoCK and ROI, TARGET-seq, GoT-seq and GoT-Splice are higher as more than one library needs to be sequenced to obtain information on the targets and transcriptome. ** Cost (in CHF) based on online quote for following kits (divided by 4 since they are for four kits): Onco-BC panel (633752): 326.00, Targeted mRNA and AbSeq Amplification Kit (633774): 1,620.00. *** Conversion AUD to CHF: conversion 1 AUD to 0.57 CHF**.** **** Conversion pounds to CHF: 1 pound to 1.11 CHF**.** ***** Conversion USD to CHF: 1 USD to 0.87 CHF.

	**Sequencing cos (CHF)**	**# cells**	**Standard library cost (CHF)**	**Targeting cost (CHF)**	**Total cost (CHF)**	**Cost per cell (CHF)**
**RoCK and ROI** *	2’000	10’000	BD: 1400	333.25	3’733	0.37
**DART-seq** [2]	1’000	10’000	DROP-seq: 700	44	1’744	0.17
**HyPR-seq**[3]	1’000	10’000	Custom	NA	NA	NA
**TARGET-seq**[4]**,***	2’000	1’000	Custom	NA	NA	NA
**BART-seq**[5]**,*****	1’000	10’000	Custom	NA	NA	0.87^a
**GoT-seq**[6]**,***	2’000 + 1’000 ONT	10’000	10x: 1600	NA	NA	NA
**GoT-Splice**[7]**,***	2’000 + 1’000 ONT	10’000	10x: 1600	NA	NA	NA
**Chigene**[8]	NA	NA	Custom	NA	NA	NA
**BD Targeted Amplification**[9]**,***	1’000	10’000	BD: 1400	486.50	2886.50	0.29
**RAGE-seq**[10]**,*****	1’000 + 7’000 ONT	10’000	10x: 1600	761.22[^a]	7761.22	0.88
**Van Horebeek et al.**[11]	1’000	10’000	10x: 1600	NA	NA	NA
**Constellation-seq**[12]**,******	1’000	10’000	DROPseq: 700<br>10x: 1600	DROP-seq: 221.08[^a],<br>10x: 55.27[^a]	DROP-seq: 1’921<br>10x: 2’655	DROP-seq: 0.19<br>10x: 0.27
**scTalLoR-seq**[13]	1’000 + 6’500 ONT	10’000	10x: 1600	NA	NA	NA
**scTLR-seq**[14]	1’000 + 1’000 ONT	10’000	10x: 1600	NA	NA	NA
**scCapture-seq**[15]	1’000	1’000	Multiple	NA	NA	NA

Supp Table 5: Additional targeting costs for ROCK and ROI scRNA-seq experiments.
The costs include bead modification, fluorescent assay and scRNA-seq experiment. Costs are calculated based on prices obtained in Switzerand. Cost of splints are based on quotes from Microsynth AG. *Number of samples:* number of full bead vials which can be modified with ordered product.

Reagent	Product number	Number of samples	Cost (CHF)	Cost per sample (CHF)
**T4 polymerase**	EP0062	1	210.65	210.65
**Lambda exonuclease**	M0262	2	112.60	56.30
**Tris, pH 8.0**	AM9855G	200	91.65	0.45
**EDTA, pH 8.0**	AM9260G	1’000	98.65	0.1
**Tween20**	13464259	25’000	40.32	<0.1
**dNTPs (10 mM)**	41106305	3	164	54.70
**DNAse / RNAse free ddH$_2$O**	10977015	125	42	0.30
**Splint**		10	80	8
**Fluorescent oligos (cost for positive and negative controls)**		80	165	2
**ROIseq primer (1 primer)**		1500	50	<0.1
**Indexing primer**		95	72	0.75
**Sequencing primer**		700	50	<0.1

Total for 1 sample: 333.25 CHF

Supp Table 6: Additional targeting costs for DART-seq scRNA-seq experiments.
The costs include bead modification, fluorescent assay and scRNA-seq experiment. Costs are calculated based on prices obtained in Switzerand. Cost of splints are based on quotes from Microsynth AG. *Number of samples:* number of full bead vials which can be modified with ordered product.

Reagent	Product number	Number of samples	Cost (CHF)	Cost per sample (CHF)
**T4 DNA Ligase**	EL0013	18	300.65	16.70
**ddH2O**	10977015	125	42	0.30
**0.5 M EDTA**	BDH7830-1	1’000	200	0.2
**Tris pH 7.5**	15567027	200	86.65	0.43
**10% SDS solution**	AM9823	400	385.00	0.96
**Tween20**	85115	2500	1’168.00	0.46
**Oligo 1 (splint 1)**		3 (based on 500 uL of probe)	65.18	21.72
**Oligo 2 (splint 1)**		3 (based on 500 uL of probe)	36.28	1.20
**Fluorescent oligos (cost for positive and negative controls)**		80	165	2
**T4 DNA Ligase**		18	300.65	16.70
**ddH2O**		125	42	0.30
**0.5 M EDTA**		1’000	200	0.2
**Total for 1 sample: 44 CHF**				

Supp Table 7: Sensitivity of targeting.

Values are extrapolated from reports in study. *positive cells unmodified (%):* percent of positive cells detected in the unmodified sample (when applicable). *positive cells targeted (%):* percent of positive cells detected in targeted sample. *Increase in positive cells with targeting:* average difference in percentage positive cells between targeted and unmodified sample. *Fold change on-target*: increase in detection of target compared to unmodified sample (when applicable). *^a:* values extrapolated from results in paper. *NA:* data not available. ***** values calculated based on average in Supplemental Table 2. ** calculations based on P1 and P2 in Table 2. Additional comments on Supp Table 6: **RoCKseq:** values are average for cell line experiment compared to unmodified sample; **RoCK and ROI:** values are average for cell line experiment compared to unmodified sample; **DART-seq:** fold change based on coverage. Values based on two separate experiments (fold change of on-target: cell line experiment / percent positive cells: PBMCs); **HyPR-seq:** fold change compared to 3' 10x. Range depends on targeted gene; **TARGET-seq:** based on detection of detection of 4 mutations in same cell. **BART-seq:** no comparison to other methods (no data); **GoT-Splice:** numbers relate to two different experiments. Fold change of on-target relates to the detection of junctions using long-read sequencing (90% reads were unique to long-read data); **Chigene:** no comparison to other methods. Capture percentages extracted from Figure 3k; **BD Targeted Amplification:** comparison to 10x data; **RAGE-seq:** comparison to untargeted data. Average percentage positive cells are calculated separately for three separate experiments; **scTLR-seq:** no unmodified sample to compare to.

	positive cells unmodified (%)	positive cells targeted (%)	Increase in positive cell[illegible] with targeting (%)	Fold change on-target
**RoCKseq**	6.4	98.50	92.02	379.7
**RoCK and ROI**	6.4	99.47	95.39	4345.7
**DART-seq** [2]	3	29	26	430
**HyPR-seq**[3]	NA	90	NA	30-300
**TARGET-seq**[4]	0	38.9	38.9	NA
**BART-seq**[5]	NA	NA	NA	NA
**GoT-seq**[6]	GoTseq: 1.4, Circularization GoT-seq: [illegible]	GoTseq: 88.7 Circularization GoT-seq: 24	GoTseq: 87.3 Circularization GoT-seq: 15	NA
**GoT-Splice**[7]	3	56	53%	12.3
**Chigene**[8]	NA	92.77	NA	NA
**BD Targeted Amplification**[9],*	NA	NA	NA	15.84[^a]
**RAGE-seq**[10]	NA	50.3 / 58.9 /61.9	NA	13
**Van Horebeek et al.**[11],**	3.22	96.68	93.46	103
**Constellation-seq**[12]	NA	NA	NA	83
**scTalLoR-seq**[13]	NA	NA	NA	29
**scTLR-seq**[14]	NA	NA	NA	NA
**scCapture-seq**[15]	NA	NA	NA	36

Supp Table 8: Number of targets for each scRNA-seq method.

# targeting elements: number of primers, probes or capture sequences used per experiment. Elements for different experiments are separated by a dash ("/"), while different types of targeting elements are split by an "&". *# transcripts targeted:* number of different targeted transcripts. *NA:* data not available

	# targeting elements	Type of targeting element	# transcripts targeted
**RoCK and ROI**	1 / 3 & 3 / 8	Capture sequences on beads & ROIseq primers	2 / 2
**DART-seq** [2]	6 / 1	Capture sequences on beads	1 / 1
**HyPR-seq**[3]	102 / 265 / 1,023	Probes	22 / 48 / 179
**TARGET-seq**[4]	12	PCR primers	12
**BART-seq**[5]	15	Specific primers	15
**GoT-seq**[6]	5	Specific primer	5
**GoT-Splice**[7]	2 & 4	Specific primer & PCR primers	2
**Chigene**[8]	NA	NA	NA
**BD Targeted Amplification**[9]	492	PCR primers	492
**RAGE-seq**[10]	644	Probes	25
**Van Horebeek et al.**[11]	1	PCR primers	1
**Constellation-seq**[12]	20 / 52 / 127	Linear amplification primer	20 / 52 / 127
**scTalLoR-seq**[13]	NA	Specific primers	NA
**scTLR-seq**[14]	1	Custom primer	1
**scCapture-seq**[15]	972	Specific primer	972

Supp Table 9: Detection of off-targets in scRNA-seq experiments.
NA: data not available.

Name	Can off-targets be determined?	Are off-targets reported?	Additional comments
**RoCK and ROI**	yes	yes	
**DART-seq** [2]	no	no	Not possible as captured transcripts from modified and unmodified transcripts will be in the same library
**HyPR-seq**[3]	yes	no	The probes are gene-specific, off-target are the detection of other genes when using specific primers
**TARGET-seq**[4]	yes	no	The primers are gene-specific, off-targets are the detection of other genes when using specific primers
**BART-seq**[5]	yes	no	The primers are gene-specific, off-targets are the detection of other genes when using specific primers
**GoT-seq**[6]	yes	no	The targeted library is sequenced separately or indexed with a separate RPI primer
**GoT-Splice**[7]	yes	no	The targeted library is sequenced separately or indexed with a separate RPI primer
**Chigene**[8]	NA	no	Not enough information available to determine
**BD Targeted Amplification**[9]	yes	no	The targeted information is obtained in a separate library
**RAGE-seq**[10]	yes	no	The targeted information is obtained in a separate library
**Van Horebeek et al.**[11]	yes	no	The targeted information is obtained in a separate library
**Constellation-seq**[12]	yes	yes	The targeted information is obtained in a separate library
**scTalLoR-seq**[13]	yes	no	The targeted information is obtained in a separate library
**scTLR-seq**[14]	yes	no	The targeted library is sequenced separately
**scCapture-seq**[15]	yes	no	The probes are gene-specific, off-target are the detection of other genes when using specific primers

Supp Table 10: Markers used for annotation of murine colonic cell clusters

Broad_cell_type	Cell_type	Marker
Epithelial	Epithelial	ENSMUSG00000045394.9__Epcam
Epithelial	Epithelial	ENSMUSG00000000303.12__Cdh1
Epithelial	Epithelial	ENSMUSG00000049382.10__Krt8
Epithelial	Secretory_cells_Goblet_Reg4	ENSMUSG00000025515.15__Muc2
Epithelial	Secretory_cells_Goblet_Reg4	ENSMUSG00000024029.4__Tff3
Epithelial	Secretory_cells_Goblet	ENSMUSG00000049350.6__Zg16
Epithelial	Secretory_cells_Goblet_Reg4	ENSMUSG00000020581.11__Agr2
Epithelial	Secretory_cells_Reg4	ENSMUSG00000073043.5__Atoh1
Epithelial	Secretory_cells_Reg4	ENSMUSG00000026531.4__Mptx1
Epithelial	Secretory_cells_Reg4	ENSMUSG00000027876.4__Reg4
Epithelial	Secretory cells_Reg4	ENSMUSG00000018927.3__Ccl6
Epithelial	Tuft	ENSMUSG00000009246.14__Trpm5
Epithelial	Tuft	ENSMUSG00000025432.11__Avil
Epithelial	Enteroendocrine	ENSMUSG00000021194.6__Chga
Epithelial	Enteroendocrine	ENSMUSG00000027350.8__Chgb
Epithelial	Enteroendocrine	ENSMUSG00000061762.12__Tac1
Epithelial	Enteroendocrine	ENSMUSG00000040046.14__Tph1
Epithelial	Stem_cells	ENSMUSG00000020140.15__Lgr5
Epithelial	Stem_cells	ENSMUSG00000009248.6__Ascl2
Epithelial	Stem_cells	ENSMUSG00000023886.10__Smoc2
Epithelial	Proliferating_stem_cells_and_transiently_amplifying	ENSMUSG00000031004.8__Mki67
Epithelial	Proliferating_stem_cells_and_transiently_amplifying	ENSMUSG00000000142.15__Axin2
Epithelial	Enterocytes	ENSMUSG00000079440.2__Alpi
Epithelial	Enterocytes	ENSMUSG00000023073.2__Slc10a2
Epithelial	Enterocytes	ENSMUSG00000035775.2__Krt20
Epithelial	Enterocytes	ENSMUSG00000000805.18__Car4
Epithelial	Enterocytes	ENSMUSG00000074115.5__Saa1
Epithelial	Enterocytes	ENSMUSG00000032122.15__Slc37a2
Epithelial	Enterocytes	ENSMUSG00000025467.8__Prap1
Mesenchymal	Mesenchymal	ENSMUSG00000026728.9__Vim
Mesenchymal	Mesenchymal	ENSMUSG00000001506.10__Col1a1
Mesenchymal	Mesenchymal	ENSMUSG00000029231.15__Pdgfra
Mesenchymal	Crypt_top_fibroblasts	ENSMUSG00000021994.15__Wnt5a
Mesenchymal	Crypt_top_fibroblasts	ENSMUSG00000008999.7__Bmp7
Mesenchymal	Crypt_top_fibroblasts	ENSMUSG00000051910.13__Sox6
Mesenchymal	Crypt_bottom_fibroblast_1_and_2	ENSMUSG00000010797.6__Wnt2
Mesenchymal	Crypt_bottom_fibroblast_1_and_2	ENSMUSG00000027840.5__Wnt2b
Mesenchymal	Crypt_bottom_fibroblast_1_and_2	ENSMUSG00000074934.3__Grem1
Mesenchymal	Crypt_bottom_fibroblast_1_and_2	ENSMUSG00000028339.17__Col15a1
Mesenchymal	Crypt_bottom_fibroblast_1_and_2	ENSMUSG00000022371.16__Col14a1
Mesenchymal	Crypt_bottom_fibroblast_1_and_2	ENSMUSG00000030772.6__Dkk3
Mesenchymal	Crypt_bottom_fibroblast_1	ENSMUSG00000030849.18__Fgfr2
Mesenchymal	Crypt_bottom_fibroblast_1	ENSMUSG00000036856.4__Wnt4
Mesenchymal	Crypt_bottom_fibroblast_2	ENSMUSG00000019880.10__Rspo3

Supp Table 11: primer sequences

Type	Name	Sequence	Modification	Purification	Scale	Dilution	Stock_concentration
Splint_sequences_enhanced_beads	Pdgfra_capture_exon_7	GAA GCT GTC AAC TTG CAC GAA GTC CAT ACC TAC TAC GCA TA	5_phosph	HPLC	0.2_µmol	ddH2O	100_µM
Splint_sequences_enhanced_beads	Pdgfra_capture_exon_17	GAC TTT GCT GGA TCT ATT GAG CTT CAT ACC TAC TAC GCA TA	5_phosph	HPLC	0.2_µmol	ddH2O	100_µM
Splint_sequences_enhanced_beads	Dual_tdtomato _eGFP	CGG CAT GGA CGA GCT GTA CAA GTA ACA TAC CTA CTA CGC ATA	5_phosph	HPLC	0.2_µmol	ddH2O	100_µM
Splint_sequences_enhanced_beads	eGFP	CAT GGT CCT GCT GGA GTT CGT GAC CCA TAC CTA CTA CGC ATA	5_phosph	HPLC	0.2_µmol	ddH2O	100_µM
Splint_sequences_enhanced_beads	ABL1	CTA AGC ATA ACT AAA GGT GAA AAG CCA TAC CTA CTA CGC ATA	5_phosph	HPLC	0.2_µmol	ddH2O	100_µM
Splint_sequences_enhanced_beads	FOXP3	CAT CGT AGC TGC TGG CAG CCA AGG CAT ACC TAC TAC GCA TA	5_phosph	HPLC	0.2_µmol	ddH2O	100_µM
Splint_sequences_enhanced_beads	RAB4B	CAA CAT CGT GGT CAT CCT CTG TGG CAT ACC TAC TAC GCA TA	5_phosph	HPLC	0.2_µmol	ddH2O	100_µM
Splint_sequences_enhanced_beads	SCN9A	CTT CCG TTT CAA TGC CAC ACC TGC CAT ACC TAC TAC GCA TA	5_phosph	HPLC	0.2_µmol	ddH2O	100_µM
Splint_sequences_enhanced_beads	ADRA1D	CAC TCA CTC AAG TAC CCA GCC ATC CAT ACC TAC TAC GCA TA	5_phosph	HPLC	0.2_µmol	ddH2O	100_µM
Splint_sequences_enhanced_beads	SOX9	CAG CTA CGG CAT CAG CAG CAC CGC CAT ACC TAC TAC GCA TA	5_phosph	HPLC	0.2_µmol	ddH2O	100_µM
Splint_sequences_enhanced_beads	KRT23	CAT TGT CAC AAC AGA CCT AGA ACA CAT ACC TAC TAC GCA TA	5_phosph	HPLC	0.2_µmol	ddH2O	100_µM
Splint_sequences_enhanced_beads	EHD3	CAA GAT CTG GAA GCT GGC CGA CAT CAT ACC TAC TAC GCA TA	5_phosph	HPLC	0.2_µmol	ddH2O	100_µM
Fluorescent_oligos	pdgfra_capture_exon_7	GAA GCT GTC AAC TTG CAC GA	Atto647N	HPLC	0.2_µmol	ddH2O	100_µM
Fluorescent_oligos	pdgfra_capture_exon_17	GAC TTT GCT GGA TCT ATT GA	Atto647N	HPLC	0.2_µmol	ddH2O	100_µM
Fluorescent_oligos	dual_tdtomato _eGFP	CGG CAT GGA CGA GCT GTA CA	Atto647N	HPLC	0.2_µmol	ddH2O	100_µM
Fluorescent_oligos	eGFP	CAT GGT CCT GCT GGA GTT CG	Atto647N	HPLC	0.2_µmol	ddH2O	100_µM
Fluorescent_oligos	FOXP3	CAT CGT AGC TGC TGG CAG CC	Atto647N	HPLC	0.2_µmol	ddH2O	100_µM
Fluorescent_oligos	KRT23	CAT TGT CAC AAC AGA CCT AG	Atto647N	HPLC	0.2_µmol	ddH2O	100_µM
Fluorescent_oligos	ABL1	CTA AGC ATA ACT AAA GGT GA					
Fluorescent_oligos	polyA	AAAAAAAAAAAAAAAAAA	Atto647N	HPLC	0.2_µmol	ddH2O	100_µM
Fluorescent_oligos	TSO	CAT ACC TAC TAC GCA TA	Atto647N	HPLC	0.2_µmol	ddH2O	100_µM
ROIseq_primers_HeLa	FOXP3	TCA GAC GTG TGC TCT TCC GAT CTC ATC TTC TGG AT	-	HPLC	0.2_µmol	ddH2O	100_µM
ROIseq_primers_HeLa	RAB4B	TCA GAC GTG TGC TCT TCC GAT CTA CAC AAT CGG CG	-	HPLC	0.2_µmol	ddH2O	100_µM
ROIseq_primers_HeLa	SCN9A	TCA GAC GTG TGC TCT TCC GAT CTA CAA CGC ATT GC	-	HPLC	0.2_µmol	ddH2O	100_µM
ROIseq_primers_HeLa	ADRA1D	TCA GAC GTG TGC TCT TCC GAT CTT GTC ATC CTC TC	-	HPLC	0.2_µmol	ddH2O	100_µM
ROIseq_primers_HeLa	SOX9	TCA GAC GTG TGC TCT TCC GAT CTC GAC GTG CAG CC	-	HPLC	0.2_µmol	ddH2O	100_µM
ROIseq_primers_HeLa	KRT23	TCA GAC GTG TGC TCT TCC GAT CTG ATG ACT TCA AC	-	HPLC	0.2_µmol	ddH2O	100_µM
ROIseq_primers_HeLa	EHD3	TCA GAC GTG TGC TCT TCC GAT CTG GAG AAG GTA TC	-	HPLC	0.2_µmol	ddH2O	100_µM
ROIseq_primers_BCR::ABL1	minor_e1a2	TCA GAC GTG TGC TCT TCC GAT CTC CTT CCA TGG AG	-	HPLC	0.2_µmol	ddH2O	100_µM
ROIseq_primers_BCR::ABL1	major_e14a2	TCA GAC GTG TGC TCT TCC GAT CTA CCA TCA ATA AG	-	HPLC	0.2_µmol	ddH2O	100_µM
ROIseq_primers_Pdgfra	roi_16	TCA GAC GTG TGC TCT TCC GAT CTA GAA ATC CAT GC	-	HPLC	0.2_µmol	ddH2O	100_µM
ROIseq_primers_Pdgfra	roi_15	TCA GAC GTG TGC TCT TCC GAT CTC GAG AGC ACA AG	-	HPLC	0.2_µmol	ddH2O	100_µM
ROIseq_primers_Pdgfra	roi_14	TCA GAC GTG TGC TCT TCC GAT CTC TGC ACC AAG TC	-	HPLC	0.2_µmol	ddH2O	100_µM
ROIseq_primers_Pdgfra	roi_13	TCA GAC GTG TGC TCT TCC GAT CTG TGA AGA TGC TC	-	HPLC	0.2_µmol	ddH2O	100_µM
ROIseq_primers_Pdgfra	roi_6	TCA GAC GTG TGC TCT TCC GAT CTC CAT TTC TGT CC	-	HPLC	0.2_µmol	ddH2O	100_µM
ROIseq_primers_Pdgfra	roi_5	TCA GAC GTG TGC TCT TCC GAT CTA CCC TGG AGA AG	-	HPLC	0.2_µmol	ddH2O	100_µM
ROIseq_primers_Pdgfra	roi_4	TCA GAC GTG TGC TCT TCC GAT CTG TTT ATG CCT TG	-	HPLC	0.2_µmol	ddH2O	100_µM
ROIseq_primers_egfp	eGFP	TCA GAC GTG TGC TCT TCC GAT CTG GCA ACT ACA AG	-	HPLC	0.2_µmol	ddH2O	100_µM
ROIseq_primers_tdtom	tdTomato_1	TCA GAC GTG TGC TCT TCC GAT CTG GCT TCA AGT GG	-	HPLC	0.2_µmol	ddH2O	100_µM
ROIseq_primers_tdtom	tdTomato_2	TCA GAC GTG TGC TCT TCC GAT CTA TGG GCT GGG AG	-	HPLC	0.2_µmol	ddH2O	100_µM
Additional_primers	T_primer	ACA GGA AAC TCA TGG TGC GT	-	HPLC	0.2_µmol	DNA_resuspension_buffer	100_µM
Additional_primers	T_primer_plus_adapter	AAT GAT ACG GCG ACC ACC GAG ATC TAC ACT CTT TCC CTA CAC ACA GGA AAC TCA TGG TGC GT	-	IEX _HPLC	0.2_µmol	DNA_resuspension_buffer	100_µM
Additional_primers	sequencing_primer	ACA CTC TTT CCC TAC ACA CAG GAA ACT CAT GGT GCG T	-	HPLC	0.2_µmol	ddH2O	100_µM
Additional_primers	polyA_protective_oligo	AAAAAAAAAAAAAAAAAA	5_phosph	HPLC	0.2_µmol	ddH2O	100_µM
Additional_primers	TSO_protective_oligo	CATACCTACTACGCATA	5_phosph	HPLC	0.2_µmol	ddH2O	100_µM

References for Supplementary Note 1 and Supplementary Tables

1. Battich, N., Stoeger, T. & Pelkmans, L. Image-based transcriptomics in thousands of single human cells at single-molecule resolution. *Nat Methods* **10**, 1127–1133 (2013).
2. Saikia, M. *et al.* Simultaneous multiplexed amplicon sequencing and transcriptome profiling in single cells. *Nat Methods* **16**, 59–62 (2019).
3. Marshall, J. L. *et al.* HyPR-seq: Single-cell quantification of chosen RNAs via hybridization and sequencing of DNA probes. *Proc. Natl. Acad. Sci. U.S.A.* **117**, 33404–33413 (2020).
4. Rodriguez-Meira, A. *et al.* Unravelling Intratumoral Heterogeneity through High-Sensitivity Single-Cell Mutational Analysis and Parallel RNA Sequencing. *Molecular Cell* **73**, 1292-1305.e8 (2019).
5. Uzbas, F. *et al.* BART-Seq: cost-effective massively parallelized targeted sequencing for genomics, transcriptomics, and single-cell analysis. *Genome Biol* **20**, 155 (2019).
6. Nam, A. S. *et al.* Somatic mutations and cell identity linked by Genotyping of Transcriptomes. *Nature* **571**, 355–360 (2019).
7. Cortés-López, M. *et al.* Single-cell multi-omics defines the cell-type-specific impact of splicing aberrations in human hematopoietic clonal outgrowths. *Cell Stem Cell* **30**, 1262-1281.e8 (2023).
8. Zhong, W. *et al.* Identification of Multi-landscape and Cell Interactions in the Tumor Microenvironment through High-Coverage Single-Cell Sequencing. Preprint at https://doi.org/10.1101/2024.06.18.599463 (2024).
9. Mair, F. *et al.* A Targeted Multi-omic Analysis Approach Measures Protein Expression and Low-Abundance Transcripts on the Single-Cell Level. *Cell Reports* **31**, 107499 (2020).
10. Singh, M. *et al.* High-throughput targeted long-read single cell sequencing reveals the clonal and transcriptional landscape of lymphocytes. *Nat Commun* **10**, 3120 (2019).
11. Van Horebeek, L. *et al.* A targeted sequencing extension for transcript genotyping in single-cell transcriptomics. *Life Sci. Alliance* **6**, e202301971 (2023).
12. Vallejo, A. F. *et al.* Resolving cellular systems by ultra-sensitive and economical single-cell transcriptome filtering. *iScience* **24**, 102147 (2021).
13. Byrne, A. *et al.* Single-cell long-read targeted sequencing reveals transcriptional variation in ovarian cancer. Preprint at https://doi.org/10.1101/2023.07.17.549422 (2023).
14. Chen, C. *et al.* Single-cell multiomics reveals increased plasticity, resistant populations, and stem-cell–like blasts in KMT2A-rearranged leukemia.
15. Pokhilko, A. *et al.* Targeted single-cell RNA sequencing of transcription factors enhances the identification of cell types and trajectories. *Genome Res.* **31**, 1069–1081 (2021).
16. Zhang, X. *et al.* Comparative Analysis of Droplet-Based Ultra-High-Throughput Single-Cell RNA-Seq Systems. *Molecular Cell* **73**, 130-142.e5 (2019).
17. Gao, C., Zhang, M. & Chen, L. The Comparison of Two Single-cell Sequencing Platforms: BD Rhapsody and 10x Genomics Chromium. *CG* **21**, 602–609 (2020).
