# Supplementary material for: RoCK and ROI: single-cell transcriptomics with multiplexed enrichment of selected transcripts and region-specific sequencing
Source: Nat Commun. 2025 Dec 10;16:10991. doi: 10.1038/s41467-025-66248-z (PMC12695968; doi:10.1038/s41467-025-66248-z)
Supplement: Supplementary file 2 — Description of Additional Supplementary Information [file 41467_2025_66248_MOESM2_ESM.pdf]

Description of Additional Supplementary Files

File Name: Supplementary Data 1
Description: Excel sheet containing information on scRNA-seq experiments with relevant metrics including sequencing depth and number of cells before and after filtering.

File Name: Supplementary Data 2
Description: Excel sheet containing information on primer sequences.
