# Supplementary material for: RoCK and ROI: single-cell transcriptomics with multiplexed enrichment of selected transcripts and region-specific sequencing
Source: Nat Commun. 2025 Dec 10;16:10991. doi: 10.1038/s41467-025-66248-z (PMC12695968; doi:10.1038/s41467-025-66248-z)
Supplement: Supplementary file 5 — Reporting Summary [file 41467_2025_66248_MOESM5_ESM.pdf]

nature portfolio

Corresponding author(s): Erich Brunner, Izaskun Mallona

Last updated by author(s): 22nd of October 2025

Reporting Summary

Nature Portfolio wishes to improve the reproducibility of the work that we publish. This form provides structure for consistency and transparency in reporting. For further information on Nature Portfolio policies, see our Editorial Policies and the Editorial Policy Checklist.

Statistics

For all statistical analyses, confirm that the following items are present in the figure legend, table legend, main text, or Methods section.

n/a	Confirmed	
☐	☒	The exact sample size (*n*) for each experimental group/condition, given as a discrete number and unit of measurement
☐	☒	A statement on whether measurements were taken from distinct samples or whether the same sample was measured repeatedly
☐	☒	The statistical test(s) used AND whether they are one- or two-sided<br>*Only common tests should be described solely by name; describe more complex techniques in the Methods section.*
☐	☒	A description of all covariates tested
☐	☒	A description of any assumptions or corrections, such as tests of normality and adjustment for multiple comparisons
☐	☒	A full description of the statistical parameters including central tendency (e.g. means) or other basic estimates (e.g. regression coefficient) AND variation (e.g. standard deviation) or associated estimates of uncertainty (e.g. confidence intervals)
☐	☒	For null hypothesis testing, the test statistic (e.g. *F*, *t*, *r*) with confidence intervals, effect sizes, degrees of freedom and *P* value noted<br>*Give P values as exact values whenever suitable.*
☒	☐	For Bayesian analysis, information on the choice of priors and Markov chain Monte Carlo settings
☒	☐	For hierarchical and complex designs, identification of the appropriate level for tests and full reporting of outcomes
☐	☒	Estimates of effect sizes (e.g. Cohen's *d*, Pearson's *r*), indicating how they were calculated

Our web collection on statistics for biologists contains articles on many of the points above.

Software and code

Policy information about availability of computer code

Data collection	Raw sequencing reads from scRNAseq experiments were processed to count tables with our novel data processing pipeline, available at https://github.com/imallona/rock_roi_method (zenodo https://zenodo.org/records/11070200). The workflow generates a transcriptome index to match the experimental design (i.e., taking the cDNA read length into account). After indexing with STAR, FASTQ files are aligned and counted using STARsolo while extracting valid cell barcodes and producing count tables for TSO and WTA readouts separately. We provide other running modes to deal with ad-hoc use cases, such as targeting repetitive sequences and hence including multimapping reads. Aside from producing count tables, our workflow generates basic scRNAseq analysis reports, including quality control and cell clustering. The generated gene by cell matrices were used for downstream quality control and analysis.<br><br>To process the mouse and human mixing experiments, a combined genome generated by concatenating GRCm38.p6 (mouse), GRCh38.p13 (human), eGFP, and tdTomato was used. For gene annotation, we used GENCODE's M25 (mouse) and v38 basic (human) and custom GTFs for eGFP and tdTomato. The data from the Pdgfrα experiment were mapped using the mouse genome GRCm38.p6and GENCODE's M25 annotation, as well as the sequence for the H2B-eGFP construct in the transgenic mouse strain.<br><br>In addition to using our novel data analysis pipeline, for the detection of fusion transcripts cDNA reads were first of all trimmed to remove sequences 5' to the ROI primer while keeping the ROI primer sequence itself, and re-aligned these to ChimerDB 4.059 (a curated gene fusion database) using STAR with disabled splicing and soft-clipping (i.e., running an end-to-end mapping that ensured alignment in both the BCR and ABL1 sides of the read). We reported alignments to minor and major BCR::ABL1 fusions (as annotated by ChimerDBv4) on cDNA reads paired to valid (e.g., as in the WTA) cell barcodes and overlapping the fusion breakpoint.
Data analysis	Analyses were run with R (version 4.3.2). Plots on the FACS analysis of barcoded beads were made primarily using the flowCore (version 2.14.0), flowViz (version 1.66.0), ggcyto (version 1.30.0) and ggplot2 (version 3.4.4) packages. Data wrangling on gene by cell matrices was

mainly performed using dplyr v1.1.4 and reshape2 v1.4.4. Plots were then generated with ggplot2 v3.4.4 and ggrastr v1.0.2. Omics downstream analyses were run mainly using the Bioconductor ecosystem: scran v1.30.2, scuttle v1.12.0, scDblFinder v1.16.0, Gviz v1.46.1, GenomicRanges v1.54.1, GenomicAlignments v1.38.2, GenomicFeatures v1.54.3 and edgeR v4.0.16. Alignment statistics were retrieved with Qualimap2 v2.3.For the BCR::ABL1 experiment, dimensionality reduction was performed with the Seurat (version 5.1.0) and SeuratObject (version 5.0.2) package using WTA-based data after quality control (including doublet removal). The Matrix package was updated to version 1.6.4 for this analysis. 2000 genes were used to find variable features using the FindVariableFeatures() function with the vst method. The FindNeighbors() function was run using 15 dimensions and the RunUMAP() function with 20 dimensions. Sankey plots were generated using SankeyMATIC (https://sankeymatic.com/, commit 088a339).

All code for downstream processing is available at https://github.com/imallona/rock_roi_paper (zenodo https://zenodo.org/records/17356998).

Images from the RNAScope experiment were analyzed with MATLAB (R2021b) and its image processing toolbox. Cell segmentation was performed using the maximum-projected Succinimidyl ester staining channel and cellpose using cyto2 model and a cell diameter of 200 pixels. Images were processed with ImageJ (Fiji version 2.0.0-rc-69/1.52p).

For manuscripts utilizing custom algorithms or software that are central to the research but not yet described in published literature, software must be made available to editors and reviewers. We strongly encourage code deposition in a community repository (e.g. GitHub). See the Nature Portfolio guidelines for submitting code & software for further information.

Data

Policy information about availability of data

All manuscripts must include a data availability statement. This statement should provide the following information, where applicable:

- Accession codes, unique identifiers, or web links for publicly available datasets
- A description of any restrictions on data availability
- For clinical datasets or third party data, please ensure that the statement adheres to our policy

Raw and processed data are available at GEO accession GSE266161. The raw RNA-sequencing data for the patient sample is deposited on EGA (EGAD50000001976 / EGAS50000001366).

Research involving human participants, their data, or biological material

Policy information about studies with human participants or human data. See also policy information about sex, gender (identity/presentation), and sexual orientation and race, ethnicity and racism.

Reporting on sex and gender	NA, these are anonymized samples and the researchers do not have access to further patient information due to data privacy and ethic regulations.
Reporting on race, ethnicity, or other socially relevant groupings	NA, these are anonymized samples and the researchers do not have access to further patient information due to data privacy and ethic regulations.
Population characteristics	NA, these are anonymized samples and the researchers do not have access to further patient information due to data privacy and ethic regulations.
Recruitment	Leftover material from routine diagnostic procedures carried out at Hämatologie Labor Kiel, University Hospital, Kiel, Germany was used for analysis of patient samples. Samples were pseudonymized and patients gave written consent to this use.
Ethics oversight	Ethical approval was obtained for Ethics committee of Kiel University, Kiel, Germany (AZ: D416/21).

Note that full information on the approval of the study protocol must also be provided in the manuscript.

Field-specific reporting

Please select the one below that is the best fit for your research. If you are not sure, read the appropriate sections before making your selection.

[x] Life sciences [ ] Behavioural & social sciences [ ] Ecological, evolutionary & environmental sciences

For a reference copy of the document with all sections, see nature.com/documents/nr-reporting-summary-flat.pdf

Life sciences study design

All studies must disclose on these points even when the disclosure is negative.

Sample size	No statistical methods were used to predetermine sample size. Experiments were all performed across thousands of cells. Instead of measuring replicates across samples, the method itself was validated across distinct cell lines, murine tissue and a human sample. The RNAScope experiment was performed on two replicates of eGFP-expressing cells. The experiments were not randomized. The investigators were not blinded to allocation during experiments and outcome assessment. Sex was not considered a factor in study design, as RoCK and ROI has no gender-specific components. The RoCK and ROI method was reproducible across samples and independent experiments. Low quality cells (determined based on detected number of genes, transcripts and mitochondrial content) were excluded after sequencing. The thresholds for exclusion were specific to the analyzed conditions and are specified in the Methods section. Measurements were taken across a

	substantial number of individual beads (approximately 1,000 per condition) to assess variability in fluorescent signal between beads. In scRNA-seq experiments, multiple independent experiments were conducted with cell numbers consistent with standard usage on the BD Rhapsody platform. These experimental designs ensured sufficient cell numbers to robustly assess methodological performance, including variability at the single-cell level, while maintaining adequate read depth per cell.
Data exclusions	scRNA-seq data were filtered for total number of counts and percent mitochondrial transcripts detected in the dT-based data. Additionally, doublets were removed. Relevant parameters are listed in the Methods section.
Replication	Experiments were all performed across thousands of cells of different types. The method itself was validated across cell lines, murine tissue and a human sample. The RNAScope experiment was performed on two replicates of eGFP-expressing cells.
Randomization	As in the BD Rhapsody platform cells are randomly deposited in the cartridge by Poisson distribution, no additional randomization was needed.
Blinding	Blinding was not necessary for this study, as knowing which samples were processes is needed to assess the method and parameters being tested.

Reporting for specific materials, systems and methods

We require information from authors about some types of materials, experimental systems and methods used in many studies. Here, indicate whether each material, system or method listed is relevant to your study. If you are not sure if a list item applies to your research, read the appropriate section before selecting a response.

Materials & experimental systems

n/a	Involved in the study
☐	☒ Antibodies
☐	☒ Eukaryotic cell lines
☒	☐ Palaeontology and archaeology
☐	☒ Animals and other organisms
☒	☐ Clinical data
☒	☐ Dual use research of concern
☒	☐ Plants

Methods

n/a	Involved in the study
☒	☐ ChIP-seq
☐	☒ Flow cytometry
☒	☐ MRI-based neuroimaging

Antibodies

Antibodies used	The CD326(EpCAM)-PE-Cy5 (eBioscience/Thermofisher, Switzerland, LOT 23658855, clone G8.8) was used for sorting of epithelial cells for the Pdgfra scRNA-seq experiment. The antibody was diluted 1:500 for use.<br><br>For sorting of human patient sample the following antibodies were used:<br><br>CD45 (V500), 5 µL, dilution 1:20, catalogue 655873, BD Horizon, clone 2D1.<br><br>CD235a (BV421), 5 µL, dilution 1:20, catalogue 562938, BD Horizon, clone GA-R2(HIR2).<br><br>Viability dye (FITC), 1 µL, dilution 1:100, catalogue 130-135-318, Miltenyi Biotec.<br><br>CD19 (PE-Cy7), 5 µL, dilution 1:20, catalogue IM3628, Beckman Coulter, clone J3.119.<br><br>CD10 (APC-Fire750), 1 µL, dilution 1:100, catalogue 312229, BioLegend, clone HI10a.<br><br>CD13 (PE), 3.5 µL, dilution 1:29, catalogue 347406, BD, clone L138.<br><br>CD33 (APC), 5 µL, dilution 1:20, catalogue 345800, BD, clone P67.6.<br><br>CD34 (BV605), 2 µL, dilution 1:50, catalogue 745247, BD OptiBuild Biosciences, clone 8G12.<br><br>CD3 (BV711), 6 µL, dilution 1:17, catalogue 344838, BioLegend, clone SK7.<br><br>BSB, 10 µL, catalogue not applicable.
Validation	All antibodies are well established and were validated by the manufacturer. All antibodies have been used and reported in prior studies.

Eukaryotic cell lines

Policy information about cell lines and Sex and Gender in Research

Cell line source(s)	L-cells and HEK293-T cells were ordered from ATCC (catalogue numbers: CRL-2648 for L-cells, CRL-3216 for HEK293-T). Insertion of eGFP and tdTomato transgenes was performed in the laboratory of Prof. Dr. Konrad Basler, University of Zurich, by lentiviral transduction and distinct clonal cell lines were established.<br>HeLa cells were kindly provided from the lab of Prof. Dr. Lukas Pelkmans, University of Zurich.
Authentication	Fluorescent signal deriving from integration of eGFP and tdTomato in cell lines was confirmed visually under a microscope. The cell lines utilized in this study were obtained from established laboratory stocks. No additional authentication procedures, such as short tandem repeat (STR) profiling, karyotyping, DNA barcoding, or species-specific PCR assays, were performed on these cell lines prior to or during the course of the experiments. Therefore, the identity and purity of these cell lines were not independently verified for this study.
Mycoplasma contamination	Cell lines were not tested for mycoplasma.
Commonly misidentified lines<br>(See ICLAC register)	No commonly misidentified cell lines were used in this study.

Animals and other research organisms

Policy information about studies involving animals; ARRIVE guidelines recommended for reporting animal research, and Sex and Gender in Research

Laboratory animals	Mice from the PdgfrαH2BeGFP strain51 were purchased from Jackson Laboratories, United States of America (strain number 007669).<br><br>Mice in the Pdgfrα scRNA-seq experiment were three males aged 2 months and 12 days (for two mice) and 1 month and 22 days.<br><br>Mice were housed in IVC (individually ventilated cages -T2 type, with plastic shelters inside, plus enrichment). The housing was under standard conditions, with 12 hours day/night cycle. The temperature in the mouse house is kept at 22°C and humidity at 50%.
Wild animals	This study did not involve wild animals.
Reporting on sex	Sex was not considered a factor in this study design, as RoCK and ROI does not have gender specific components. Only male mice were used for this study.
Field-collected samples	This study did not involve field-collected samples.
Ethics oversight	All animal based experimental procedures at the University of Zurich were performed in accordance with Swiss Federal regulations and approved by the Cantonal Veterinary Office (license ZH045/2019).

Note that full information on the approval of the study protocol must also be provided in the manuscript.

Plants

Seed stocks	No plants were used in this study.
Novel plant genotypes	No plants were used in this study.
Authentication	No plants were used in this study.

Flow Cytometry

Plots

Confirm that:

- [x] The axis labels state the marker and fluorochrome used (e.g. CD4-FITC).
- [x] The axis scales are clearly visible. Include numbers along axes only for bottom left plot of group (a 'group' is an analysis of identical markers).
- [x] All plots are contour plots with outliers or pseudocolor plots.
- [x] A numerical value for number of cells or percentage (with statistics) is provided.

Methodology

Sample preparation	Bone marrow aspirates were enriched for mononuclear cells by Ficoll density gradient centrifugation prior to cell preservation by freezing. Frozen bone marrow samples were defrosted, washed twice in PBS and cells were counted with Trypan blue to assess sample quality. If the viability was below 75%, the cells underwent dead cell removal using magnetic bead-based dead cell removal (Dead Cell Removal Kit, Miltenyi Biotec) which was performed following the manufacturer's recommendations. Part of the sample was stained with fluorescent antibodies for enriching non-leukemic live cells by FACS sorting. A list of fluorescent antibodies used for sorting is available in the Methods section of the paper. Bone marrow sample was stained firstly with the viability dye for 10 minutes on ice, washed once with PBS and stained with the antibody mix for 30 minutes at room temperature in darkness. After staining the cells were washed once in PBS and resuspended in PBS for FACS sorting.<br>For the Pdgfra-scRNAseq experiment, colonic tissues were obtained from PdgfrαH2BeGFP reporter mice. The tissues were flushed with PBS, longitudinally opened and finely minced into 2 mm pieces. Minced tissue fragments were washed with PBS three times. Following the methodology outlined by Brügger et al., 2020, tissue pieces underwent rounds of digestion to separate epithelial and mesenchymal fractions. For the detachment of the epithelial fraction, the tissue pieces were incubated in Gentle Cell Dissociation Reagent (STEMCELL Technologies, Germany) while gently rocking for 30 minutes at room temperature. The pieces were pipetted up and down for the epithelial fraction to be detached. The epithelial fraction was then filtered through a Falcon 70-µm cell strainer (Corning, Switzerland), washed with plain ADMEM/F12 and incubated for 5 minutes at 37°C in prewarmed TrypLE express (Gibco, Thermofisher, Switzerland). The gentleMACS Octo Dissociator (Miltenyi Biotec, Switzerland) m_intestine program was employed for single- cell dissociation. The obtained epithelial single-cell suspension was then filtered through a Falcon 40-µm cell strainer (Corning) and kept on ice in ADMEM/F12 supplemented with 10% FBS. For dissociation of the mesenchymal fraction, the remaining tissue pieces (following epithelium detachment) were digested for 1 hour at 37°C under 110 rpm shaking conditions in DMEM supplemented with 2 mg/mL collagenase D (Roche) and 0.4 mg/mL Dispase (Gibco). The mesenchymal fraction was then filtered through a Falcon 70-µm cell strainer (Corning), washed with plain ADMEM/F12, and subsequently filtered through a Falcon 40-µm cell strainer (Corning). The epithelial and mesenchymal cells were mixed and stained for 30 minutes on ice with anti- CD326(EpCAM)-PE-Cy5 (1:500, eBioscience/Thermofisher, Switzerland) in PBS. Prior to cell sorting, all cells were stained for 5 minutes on ice with DAPI in PBS (1:1000, ThermoFisher, Switzerland). Epithelial and mesenchymal cells labeled with PE-Cy5 and eGFP were sorted separately and subsequently mixed in a 1:1 ratio.<br>To test RoCKseq bead modification, barcoded beads were incubated with multiple fluorescent oligos acting either as positive and negative controls or specific for the modification. RoCKseq modified beads and unmodified beads used for controls were incubated with fluorescent oligos for 30 minutes at 46o C in BD Rhapsody lysis buffer (part number 650000064, BD RhapsodyTM Enhanced Cartridge Reagent Kit, BD 664887) with 1 M DTT (part number 650000063,BD RhapsodyTM Enhanced Cartridge Reagent Kit, BD 664887). An in-depth protocol is available at dx.doi.org/10.17504/protocols.io.rm7vzjyb5lx1/v1.
Instrument	Bone marrow aspirates were sorted on a BD FACS Aria III Fusion (BD Biosciences, Germany).<br>Murine cells for the Pdgfra-scRNAseq experiment were sorted on a FACS Aria III (BD Biosciences, Switzerland).<br>Fluorescent signal from barcoded beads was analyzed on a FACS Canto II 2L with HTS (BD Biosciences, Switzerland).
Software	The BD FACSDiva software was used for data acquisition. Analysis of .fcs files for barcoded beads were run with R (version 4.3.2). Plots on the FACS analysis of barcoded beads were made primarily using the flowCore (version 2.14.0), flowViz (version 1.66.0), ggcyto (version 1.30.0) and ggplot2 (version 3.4.4) packages. Analysis of FACS data from the Pdgfra experiment was performed using the FCS Express software (version 6).
Cell population abundance	Viability of sorted bone marrow aspirates was >60% of gated singlets.<br>Viability of Pdgfra cells was >70% of gated singlets.
Gating strategy	For sorting of bone marrow aspirates, debris were first of all removed by gating on FSC-A vs SSC-A based on cell size. Single cells were then selected using a FSC-A vs FSC-H dot plot. Viable cells were then determined by plotting the signal from the viability dye against the SSC-A and excluding cells with a high signal deriving from the viability dye.<br>Gating on barcoded beads was performed using a FSC-A vs SSC-A plot to remove debris. Non-leukemic compartment cells were then sorted based on CD235a-CD45lowCD19-CD10- and B cell precursor cells/leukemic cells based on CD235a-CD45lowCD19+.<br>For the murine cells of the Pdgfra experiment, debris were first of all removed by gating on FSC-A vs SSC-A based on cell size. Single cells were then selected using a FSC-A vs FSC-H dot plot. Live cells (included gate) were gated on FITC-A (signal from eGFP positive cells) versus Pacific Blue-A (viability signal).<br>Barcoded beads were first of all gated on FSC-A vs SSC-A plots to remove debris. Gating was done based on bead size. No additional gating was performed on the sample.

☒ Tick this box to confirm that a figure exemplifying the gating strategy is provided in the Supplementary Information.
